# Sialidase NEU1 suppresses progression of human bladder cancer cells by inhibiting fibronectin-integrin α5β1 interaction and Akt signaling pathway

**DOI:** 10.1186/s12964-019-0500-x

**Published:** 2020-03-12

**Authors:** Xiaoman Zhou, Yanhong Zhai, Changmei Liu, Ganglong Yang, Jia Guo, Guang Li, Chengwen Sun, Xiaowei Qi, Xiang Li, Feng Guan

**Affiliations:** 1 The Key Laboratory of Carbohydrate Chemistry and Biotechnology, Ministry of Education, School of Biotechnology, Jiangnan University, Wuxi, China; 2 Department of Urology, Affiliated Hospital of Jiangnan University, Wuxi, China; 3 Department of Pathology, Affiliated Hospital of Jiangnan University, Wuxi, China; 4 Provincial Key Laboratory of Biotechnology, Joint International Research Laboratory of Glycobiology and Medicinal Chemistry, College of Life Science, Northwest University, Xi’an, China

**Keywords:** Sialic acids, Sialidase, Apoptosis, Fibronectin, Integrin

## Abstract

**Background:**

Sialic acids are widely distributed in animal tissues, and aberrantly expressed in a variety of cancer types. High expression of sialic acid contributes to tumor aggressiveness by promoting cell proliferation, migration, angiogenesis, and metastasis. Sialidases are responsible for removal of sialic acids from glycoproteins and glycolipids.

**Methods:**

N-glycomics of bladder cancer cells were detected by MALDI-TOF mass spectrometry. Sialic acid modification in bladder cancer tissue was determined by lectin blot. The down-regulation of NEU1 in bladder cancer cells was determined by high resolution liquid chromatography mass spectrometry (HR LC-MS). The effects of sialidase NEU1 expression on proliferation and apoptosis of human bladder cancer cells were examined by western blot, RT-PCR, confocal imaging and flow cytometry. Moreover, the function of sialic acids on fibronectin-integrin α5β1 interaction were assayed by immunoprecipitation and ELISA. The importance of NEU1 in tumor formation in vivo was performed using BALB/c-nu mice. Expression of NEU1 in primary human bladder cancer tissue samples was estimated using bladder cancer tissue microarray.

**Results:**

(1) Downregulation of NEU1 was primarily responsible for aberrant expression of sialic acids in bladder cancer cells. (2) Decreased NEU1 expression was correlated with bladder cancer progression. (3) NEU1 overexpression enhanced apoptosis and reduced proliferation of bladder cancer cells. (4) NEU1 disrupted FN-integrin α5β1 interaction and deactivated the Akt signaling pathway. (5) NEU1 significantly suppressed in vivo tumor formation in BALB/c-nu mice.

**Conclusions:**

Our data showed that NEU1 inhibited cancer cell proliferation, induced apoptosis, and suppressed tumor formation both in vitro and in vivo, by disrupting interaction of FN and integrin β1 and inhibiting the Akt signaling pathway. Our observations indicate that NEU1 is an important modulator of the malignant properties of bladder cancer cells, and is a potential therapeutic target for prognosis and treatment of bladder cancer.

Video Abstract

**Graphical abstract:**

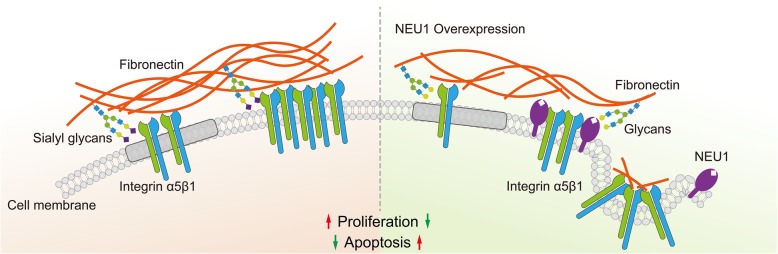

## Background

Sialic acid, a monosaccharide with a nine-carbon backbone, is often the terminal group on carbohydrate chains of glycoproteins and glycolipids [1, 2]. Sialic acids are widely distributed in animal tissues. N-acetylneuraminic acid (Neu5Ac) and N-glycolylneuraminic acid (Neu5Gc) are the two major sialic acids in mammals [3]. Because of their negative electric charge, sialic acids play crucial roles in a variety of cellular functions, e.g., cell-cell interaction, determining conformation of glycoproteins on cell membranes, and masking antigenic determinants on receptor molecules [4–11].

Epithelial-mesenchymal transition (EMT) is a biological conversion process of polarized epithelial cells to mesenchymal phenotype, characterized by loss of cell-cell adhesion and epithelial polarity as well as the acquisition of migratory and invasive properties. EMT is regulated by multiple signaling networks, including extracellular signal-regulated protein kinases (ERKs), mitogen-activated protein kinase (MAPK), phosphatidylinositol 3-kinase (PI3K)/Akt, Smads, RhoB, β-catenin and c-fos [12–15]. Other than these signaling factors, aberrant expression and high sensitivity of sialic acid as a tumor marker have been reported in a variety of cancerous conditions, and in many EMT models [16–18]. High expression of sialic acid contributes to tumor aggressiveness by promoting cell proliferation, migration, angiogenesis, and metastasis. Twenty known sialyltransferases are responsible for addition of sialic acid to glycoproteins or glycolipids [19]. Dysregulated expression of certain sialyltransferases has been correlated with abnormal sialylation in cancer cells [20]. For example, overexpression of β-galactoside α-2,6-sialyltransferase 1 (ST6GAL1) and α-2,8-sialyltransferase 2 (ST8SIA2) was associated with enhanced invasive phenotype and chemosensitivity of human hepatocellular carcinoma cells [21]. ST6GAL1 promotes transforming growth factor β (TGFβ)-dependent EMT, and also maintenance of malignant phenotype of human carcinomas through a non-Smad signaling pathway [22]. Poly sialic acid, whose production is catalyzed by polysialyltransferases ST8SIA2 and ST8SIA4, shows high aberrant expression on a variety of tumors [23].

Dysregulated expression of sialidases (also known as neuraminidases) has been observed in many types of cancer. Four mammalian sialidases (termed NEU1, − 2, − 3, − 4) have been identified to date. Each has a distinct cellular location: NEU1 in lysosomes, NEU2 in cytoplasm, NEU3 in plasma membrane, and NEU4 in mitochondria [17]. Sialidase expression and function are frequent topics in cancer research. Upregulation of NEU3 expression has been reported in colon cancer [24] and renal cell carcinoma [25]. Inhibition of NEU3 expression resulted in accumulation of ganglioside GM3 in HeLa and A549 cells, leading to reduction of epidermal growth factor receptor (EGFR)/ ERK signaling and consequent reduction of cell growth [26]. NEU1 played a crucial role in regulation of integrin β4-mediated signaling through desialylation of integrin β4, and suppressed metastasis of human colon cancer cells [27]. In human lung microvascular endothelia, NEU1 inhibited in vitro angiogenesis through desialylation of adhesion molecule CD31 [28].

Bladder cancer is a common malignancy, affecting approximately 549,393 adults and leading cause of death about 199,922 patients worldwide in 2018 [29–31]. Our previous report described global quantitative glycomics analysis of human cell lines HCV29 (normal bladder epithelia), KK47 (benign non-muscle-invasive bladder cancer), and YTS-1 (highly malignant invasive bladder cancer) using lectin microarray and mass spectrometry (MS) methods [32]. We observed the carbohydrate antigen sialyl Lewis X (sLe^x^), which is associated with tumor formation and metastasis, was significantly upregulated in bladder cancer cells, suggesting that bladder cancer formation may involve aberrant expression of sialylation.

We now describe (i) analysis of the expression of sialylated glycans and sialidases in bladder cancer cells in comparison to normal bladder epithelial cells, using quantitative glycomics analysis and SILAC (stable isotope labeling by amino acids in cell culture) technique, (ii) evaluation of aberrant NEU1 expression and its functional role in apoptosis and proliferation of bladder cancer cells, and (iii) suppression of human bladder cancer by NEU1 in vitro and in vivo.

## Methods

### Cell lines and cell culture

Human cell lines HCV29 (normal bladder mucosal epithelia), KK47 (benign non-muscle-invasive bladder cancer), and YTS-1 (highly malignant invasive bladder cancer) were kindly provided by Dr. S. Hakomori (The Biomembrane Institute, Seattle, WA, USA) [33]. Human uroepithelial cell line SV-HUC-1, transitional carcinoma cell lines T24 and J82 were from the Cell Bank of the Chinese Academy of Sciences (Shanghai). Cells were cultured as described in Supplementary Information/Methods.

### Determination of sialidase activity

Sialidase activity was determined using 2′-(4-methylumbelliferyl) -α-D-N-acetylneuraminic acid sodium (4-MU-NANA) (Sigma-Aldrich) as substrate [34]. Fluorescence intensity was measured by spectrofluorometer (Synergy H4 Hybrid Multi-Mode Microplate Reader, BioTek; Winooski, VT, USA) with excitation wavelength 365 nm and emission wavelength 450 nm.

### Derivatization of released N-linked glycans with [^12^C_6_]- or [^13^C_6_]-aniline

Ten μL [^12^C_6_]- or [^13^C_6_]-aniline (Sigma-Aldrich) and 25 μL fresh NaCNBH_3_ (1 M) prepared in DMSO/ acetic acid (7:3, v/v) were added separately to amidated N-linked glycans from KK47 and HCV29 cells, and incubated at 75 °C for 10 min as described previously [32, 35]. The mixture was lyophilized under vacuum, redissolved in 500 μL 1-butanol/ methanol/ H_2_O (BMW), and desalted with Sepharose 4B. Eluted glycan derivatives were dried and stored in the dark at − 20 °C.

### MS analysis of sialylated N-glycan profiles

N-glycans were characterized by MALDI-TOF/TOF-MS (UltrafleXtreme, Bruker Daltonics; Bremen, Germany) as described previously [32]. Representative MS spectra of N-glycans with signal-to-noise ratios > 3 were annotated using the GlycoWorkbench software program (version:2.1). Relative intensity was analyzed and generated using FlexAnalysis software (Bruker Daltonics, version:3.3). Coefficient of variation (CV) percentages based on relative intensity values were used to estimate the stability of mass spectrometry. Total sum quantitative of the sialylated N-glycans (*S*_*i*_) were calculated by the equation:

$$ {S}_i={S}_{ij}\times {N}_{ij}+{S}_{i\left(j+1\right)}\times {N}_{i\left(j+1\right)}+\dots +{S}_{i\left(j+n\right)}\times {N}_{i\left(j+n\right)} $$

with *S*_*ij*_ = relative intensity of N-glycan j in i cells, and *N*_*ij*_ = number of sialic acids of N-glycan j in i cells [32].

### FN-integrin α5β1 binding assay in vitro

Purified FN were dissolved in PBS to 50 μg/mL and coated on to ELISA plates (5 μg/cm^2^) overnight at 4 °C. The plates were washed with PBS and blocked with 3% BSA (m/v, in PBS).

Sialic acids on FN were removed by adding 1 U/mL sialidase and incubating at 37 °C for 30 min. After washing three times with PBS, the plates were incubated with integrin α5β1 (20 μg/mL, in PBST with 0.5% BSA) for 12 h at 4 °C with gentle shaking. After washing three times with PBST, the integrin α5β1 binding ratio can be detected with HRP conjugated integrin β1 antibody (1:1000) and TMB-ELISA Substrate Solution.

### Tumor formation in mice

Animal experiments were performed in accordance with the Animal Care and Use Committee guidelines of Jiangnan University. YTS-1/Ctrl and YTS-1/NEU1 cells were suspended in RPMI-1640 medium without FBS at a density of 1 × 10^7^ cells /mL, and 0.2 mL aliquots were transplanted subcutaneously into 8-week-old male BALB/c-nu mice. Tumor size was measured every other day for 21 days. At the end of 3 weeks, tumors were excised and weighed.

### Statistical analysis

All values were presented as mean ± SD from three independent experiments unless otherwise specified. Differences between means were analyzed by Student’s t-test.

## Results

### Sialoglycans are highly expressed in bladder cancer cells

**Fig. 1:**
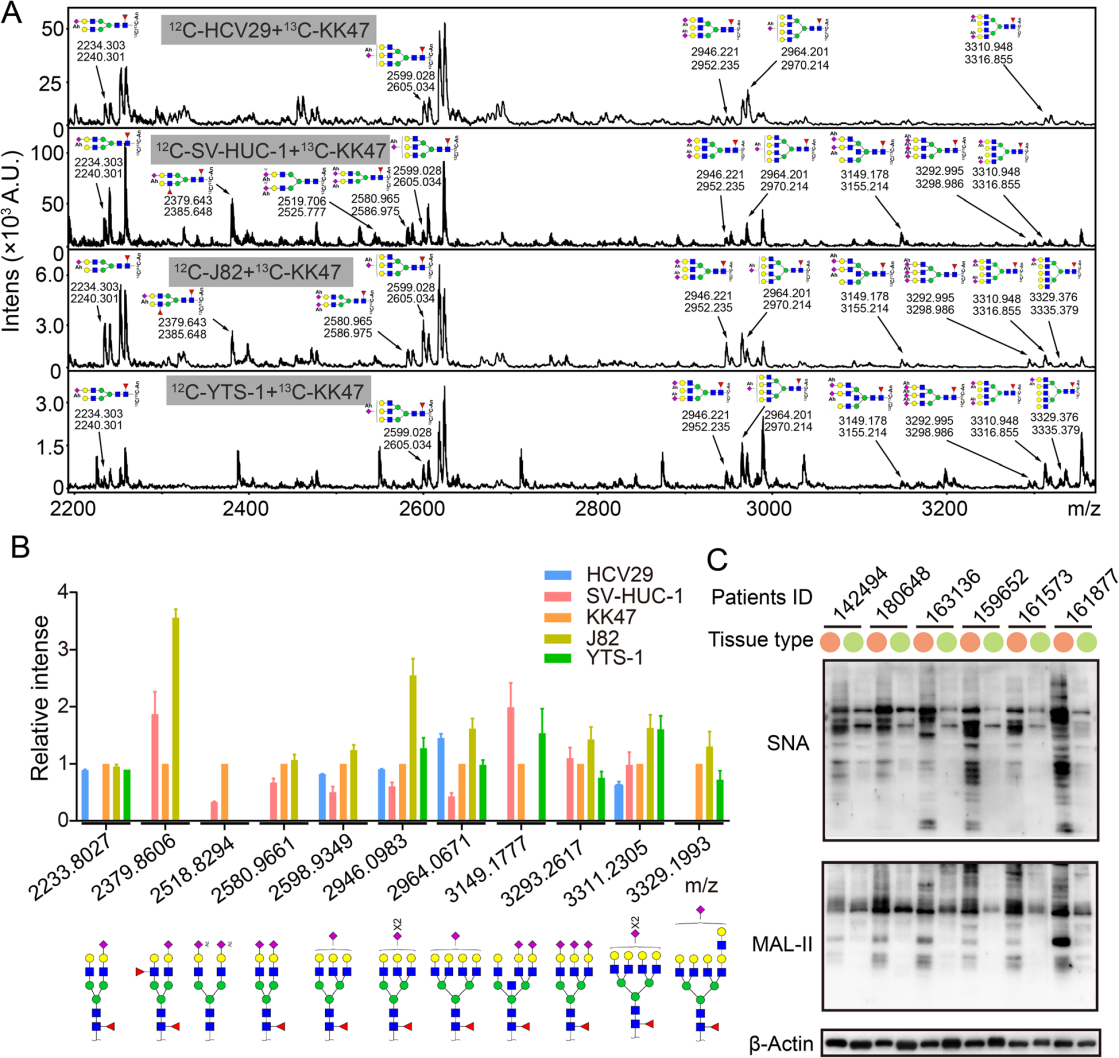
Expression of sialylated N-glycans in five bladder cancer or epithelial cell lines. N-glycans of HCV29, KK47, YTS-1, SV-HUC-1, and J82 cells were converted with acetohydrazides and released. N-glycans from KK47 cells were derivatized using [^13^C_6_]-aniline, and their expression level was defined as 1 for comparative analytical purposes. N-glycans from other cells were derivatized using [^12^C_6_]-aniline. The annotated N-glycans were detected in at least three biological replications. **a** Sialylated N-glycans in the five cell lines were annotated. **b** Relative expression of sialylated N-glycans was quantified and presented as mean ± SD from triplicate experiments. **c** Sialic acids in bladder cancer tissue (orange) and the adjacent tissue (green) form patients with bladder cancer. Lectin blot analysis was performed using biotinylated lectin SNA and MAL-II to detect sialic acids modification in clinic samples. Signals were detected using a Vectastain ABC kit (Vector Laboratories; Burlingame, CA, USA) as described in Supplementary information / Methods. β-Actin expression was used as a loading control

Sialylated N-glycans from five bladder cancer cell lines (see Methods in Supplementary Information) were derivatized using isotope tags and analyzed. Eleven sialylated N-glycans were observed as a doublet with a 6-Da difference. The identified derivatized sialylated glycans are described in Fig. 1a. Expression levels of sialylated N-glycans were normalized as described in the Fig. 1 legend, and the putative structures are shown in Fig. 1b. Levels of sialylated N-glycans in normal bladder cells were generally < 1, and were lower than levels in bladder cancer cells. Averages of the ratio of N-glycans at m/z 2580.97 (SV-HUC-1: KK47: J82 = 0.67: 1: 1.06), 2598.93 (HCV29: SV-HUC-1: KK47: J82 = 0.82: 0.50: 1: 1.24), 2946.10 (HCV29: SV-HUC-1: KK47: J82: YTS-1 = 0.91: 0.61: 1: 2.55: 1.28), and 3311.23 (HCV29: SV-HUC-1: KK47: J82: YTS-1 = 0.64: 0.98: 1: 1.63: 1.61) were significantly higher in bladder cancer cells (Table S1). Results consisted with our bladder cancer tissue sialic acids lectin blotting [32]. *Sambucus nigra* lectin (SNA, recognizing α2,6-linked sialic acids) and *Maackia amurensis* lectin (MAL-II, recognizing α2,3-linked sialic acids) were used to detect sialic acids level on tissue protein of clinic bladder cancer patients. Sialic acids were significantly rich in tumor tissue sample compared to tumor adjacent tissues (Fig. 1c, Table. S2). Indicating that, accumulated sialic acids modification existed in bladder cancer cells.

### Downregulation of NEU1 is responsible for aberrant expression of sialoglycans

**Fig. 2:**
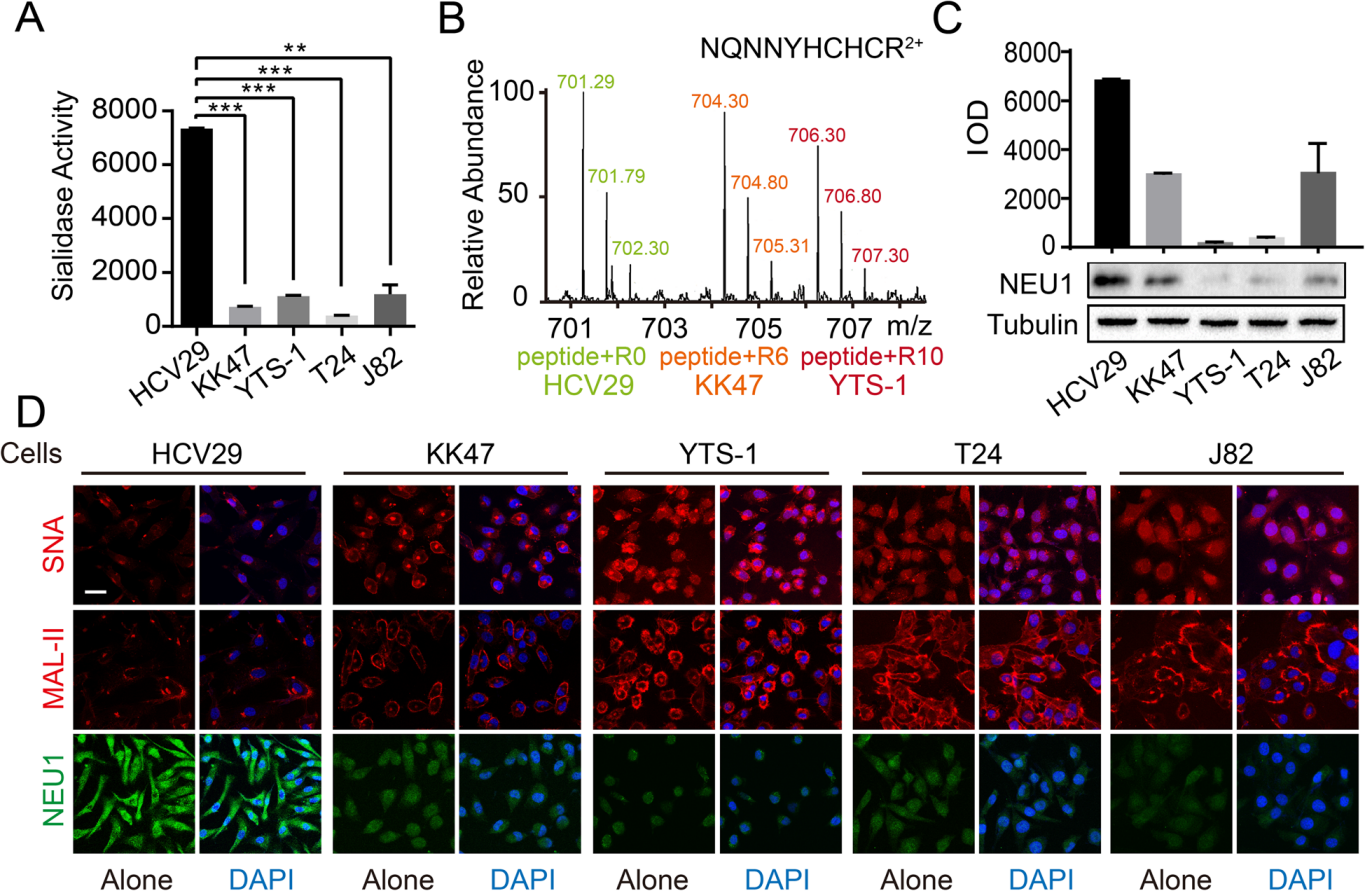
Downregulation of NEU1 in bladder cancer cells. **a** Enzymatic activities of total sialidases in five cell lines as shown were quantitatively analyzed based on fluorescence intensity, and presented as mean ± SD from triplicate experiments. **, *P* = 0.001–0.005. ***, *P* < 0.001. **b** Expression of NEU1 in HCV29, KK47 and YTS-1 by SILAC analysis. Cells were cultured in SILAC-labeled RPMI 1640 with 10% dialyzed FBS containing “light” (K0R0), “medium” (K4R6), or “heavy” (K8R10) Lys and Arg as described previously [36], and subjected to LC-MS/MS analysis. Specific peptides of NEU1 in the three cell lines were annotated. **c** Data on NEU1 in HCV29, KK47, YTS-1, T24, and J82 cells from Western blotting (left) and relative integrated optical density (IOD) were quantified using the Image J software program, and presented as mean ± SD from triplicate experiments (right). **d** Sialic acids in the five cell lines stained by Cy3-labeled lectins SNA or MAL-II (red) and merged with DAPI (blue), or by immunofluorescence staining with anti-NEU1 antibody (green) and merged with DAPI (blue). scale bar: 50 μm

In mammalian cells, sialyltransferases and sialidases are responsible (respectively) for addition and removal of sialic acids on glycans. We evaluated total sialidase activities of HCV29, KK47, YTS-1, and T24 cells using 4-MU-NANA as substrate. Sialidase activity was highest for normal bladder cell line HCV29, and significantly lower for bladder cancer cell lines KK47, YTS-1, and T24 (Fig. 2a).

Proteome levels of SILAC-labeled HCV29, KK47, and YTS-1 cells were quantitatively analyzed in our previous study [36]. Among the known ~ 20 sialyltransferases and 4 sialidases, a differential signal among these three cell lines was observed only for sialidase NEU1. HR LC-MS/MS analysis showed that higher expression of NEU1 in HCV29, and lower expression in KK47 and YTS-1 (Fig. 2b, Fig. S1). Quantitative real-time PCR and Western blotting similarly showed lower expression of NEU1 in KK47, J82, YTS-1, and T24 than in HCV29, at both mRNA (Fig. S2) and protein levels (Fig. 2c).

Fluorescence staining analysis revealed the same trend. Cells were stained separately with Cy3-labeled SNA and MAL-II lectins. SNA and MAL-II binding capacities were significantly higher for KK47, J82, YTS-1, and T24 than for HCV29 (Fig. 2d). In contrast to sialic acid expression, NEU1 expression was higher for HCV29 than for the four cancer cell lines (Fig. 2d). Taken together, these findings suggest that downregulated expression of NEU1 is responsible for the aberrant expression of sialic acids in bladder cancer, and is associated with bladder tumor progression.

### Downregulation of NEU1 and upregulation of sialic acid during EMT

**Fig. 3:**
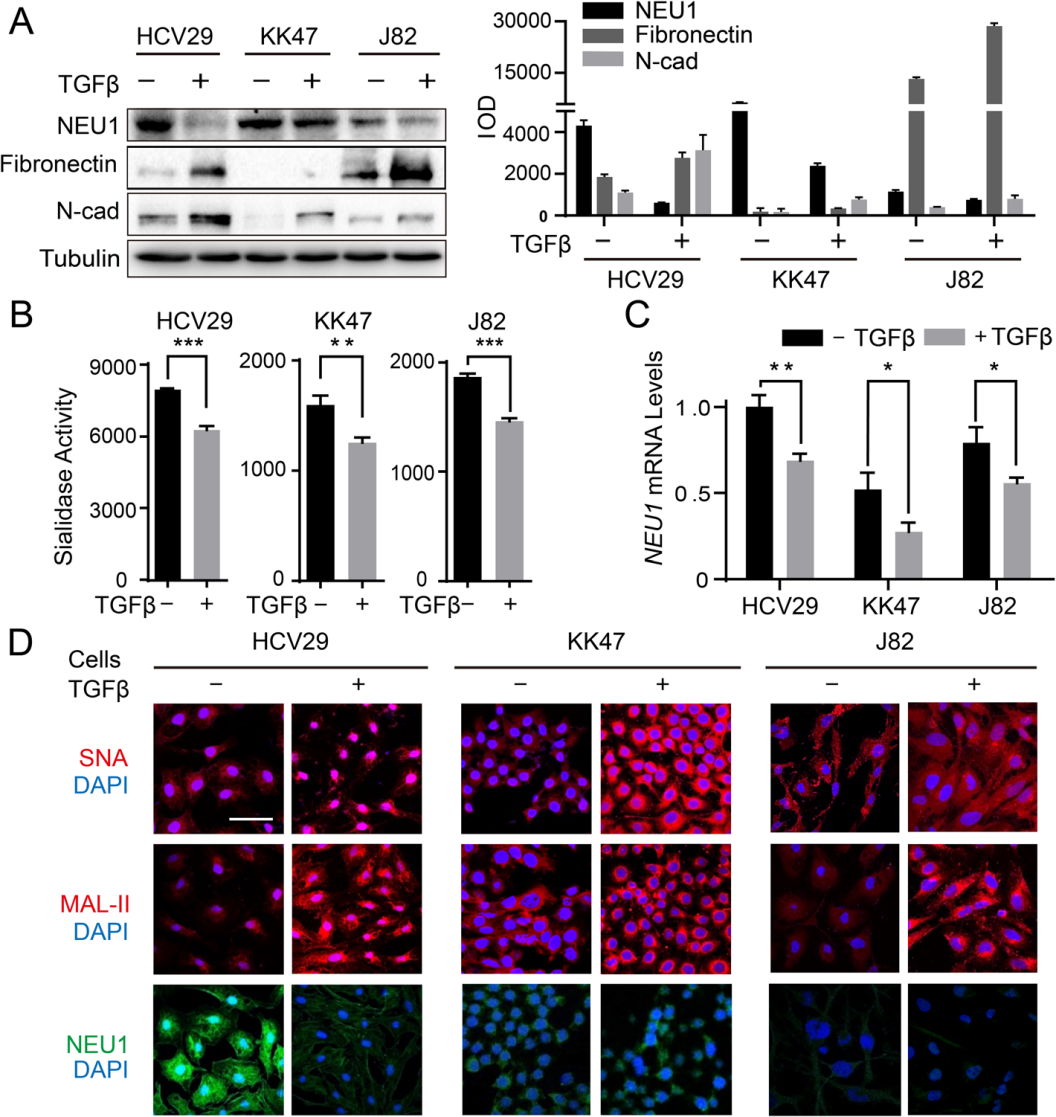
Decreased expression of NEU1 during EMT. **a** HCV29, KK47, and J82 cells were treated with 5 ng/mL TGFβ for 48 h. Total protein was extracted with RIPA buffer, subjected to SDS-PAGE, and transferred to PVDF membrane. Membranes were incubated with antibodies against NEU1, FN, and N-cadherin, incubated with appropriate HRP-conjugated secondary antibody, and proteins were revealed with Super-signal Chemiluminescence substrate kit as described in Supplementary information / Methods. β-tubulin: loading control. **b** Enzymatic activities of total sialidases in TGFβ-treated cells as described in Fig. 2A. **c** Expression of NEU1 at the mRNA level in TGFβ-treated and nontreated cells. **d** Sialic acids in TGFβ-treated and nontreated cells were stained by Cy3-labeled SNA, MAL-II (upper, middle), or by immunofluorescence staining with anti-NEU1 (lower). Scale bar: 100 μm. *, *P* = 0.01–0.05. **, *P* = 0.001–0.005. ***, *P* < 0.001

EMT, a crucial step in tumor invasion and metastasis, is a process whereby epithelial cells acquire high migratory and invasive capability, escape from the primary basement membrane to which they are normally attached, and migrate to distant sites [37]. Following TGFβ treatment, HCV29, KK47, and J82 cells showed increased levels of the typical mesenchymal markers FN and N-cadherin (Fig. 3a), and enhancement of cell motility (Fig. S3). As enzymatic activities of total sialidases decreased (Fig. 3b), surface sialylation of HCV29, KK47, and J82 (recognized by lectin MAL-II and SNA) increased (Fig. 3d). Downregulation of NEU1, observed at the protein and mRNA levels (Fig. 3a, c, d), was primarily responsible for the upregulation of sialic acid during EMT.

### NEU1 overexpression decreases cell growth and increases apoptosis

To investigate the molecular function of NEU1 in bladder cancer, we cloned the NEU1 gene, transfected it into bladder cancer cell lines YTS-1 and T24, and thus generated transfectant cell lines YTS-1/NEU1 and T24/NEU1.

**Fig. 4:**
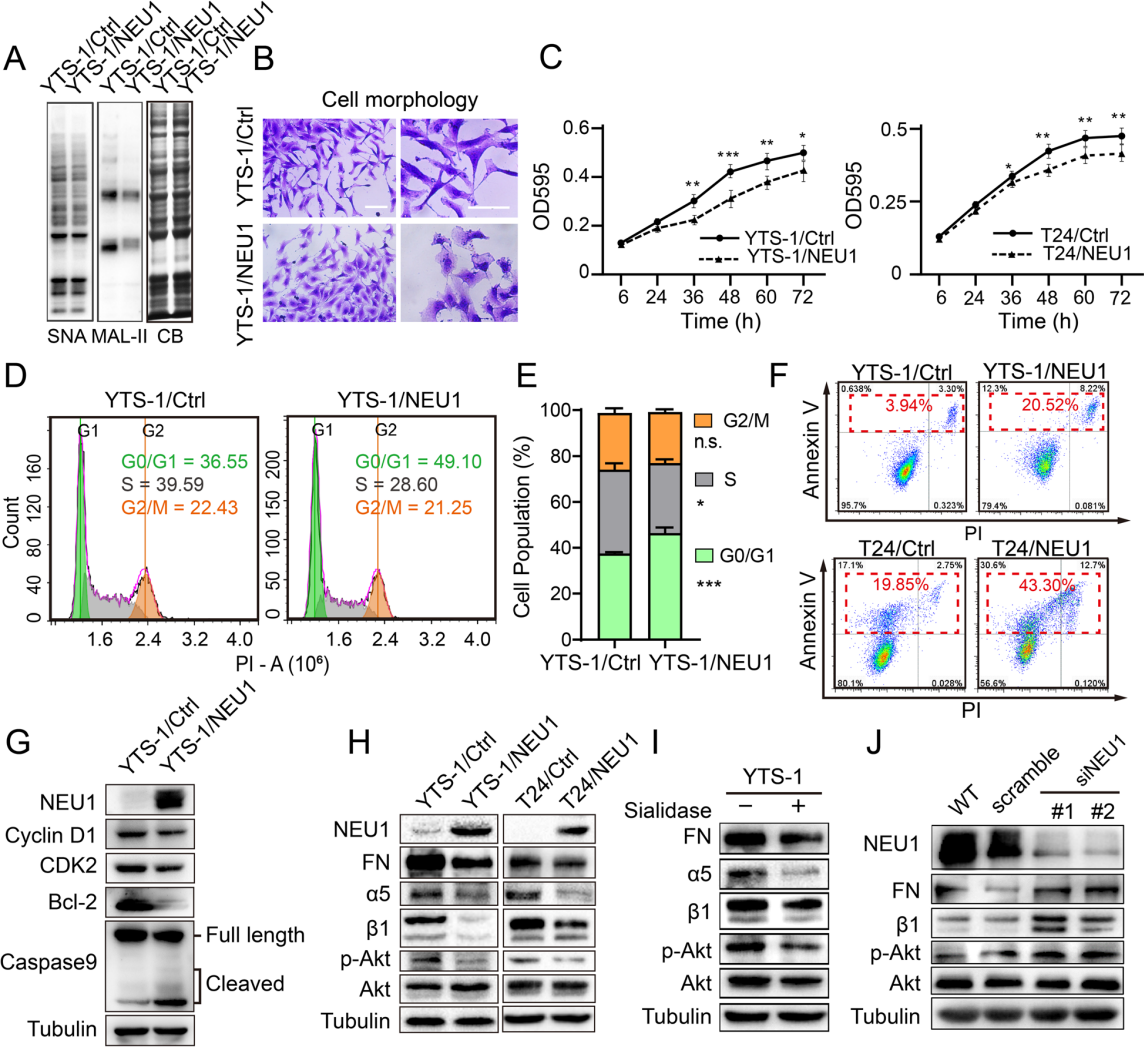
NEU1 suppresses cell proliferation and induces apoptosis by inhibiting the Akt pathway. **a** Lectin blotting assay: Total proteins from YTS-1/Ctrl and YTS-1/NEU1 were extracted and subjected to SDS-PAGE. Biotin-labeled lectin blotting was performed, and signals were detected using a Vectastain ABC kit (Vector Laboratories; Burlingame, CA, USA). Coomassie blue staining (CB) of SDS-PAGE was used as loading control. **b** Cell morphology assay: YTS-1/Ctrl and YTS-1/NEU1 cells were seeded on 6-cm plates with complete culture medium, washed with ice-cold PBS, cultured 24 h, fixed with 4% fresh polyformaldehyde, and stained by crystal violet. Scale bar: 50 μm. **c** Cell proliferation (MTT assay). YTS-1/Ctrl, YTS-1/NEU1, T24/Ctrl, and T24/NEU1 cells (4 × 10^3^/well) were seeded on 96-well plates, and cultured for 6, 24, 36, 48, 60, or 72 h. Proliferation was assessed by MTT assay, and data were analyzed by the Prism software program and presented as mean ± SD from triplicate experiments. *, *P* = 0.01–0.05. **, *P* = 0.001–0.005. ***, *P* < 0.001. **d** Cell cycle analysis of YTS-1/Ctrl and YTS-1/NEU1. Effect of NEU1 over expression on cell cycle in YTS-1 cells, as measured by PI staining and flow cytometry. **e** The quantification of cell population distribution in different stages of cell cycle. **f** Cell apoptosis (FACS analysis). Cells were cultured overnight, detached by trypsin, incubated with Annexin V-FITC and PI for 20 min in the dark, and subjected to flow cytometry. Data were analyzed using the FlowJo software program. Representative results from two independent experiments are shown. **g** Cell cycle and apoptosis markers detected by Western blotting. Cyclin D1 and CDK2 were used as cell cycle marker. Bcl-2 and Caspase9 were used as apoptosis marker. **h** Total proteins from the four cell lines were extracted and subjected to SDS-PAGE and Western blotting. Signals for NEU1, FN, α5, β1, Akt, and phosphorylated Akt were revealed using Supersignal Chemiluminescence substrate kit as described in Supplementary information / Methods. **i** YTS-1 cells were cultured in complete medium for 12 h, and incubated with sialidase NEUA3 in serum-free medium for 30 min. Expression of FN, α5, β1, Akt, and phosphorylated Akt was analyzed by Western blotting. **j** HCV29 cells were transiently transfected with scrambled siRNA (control) or with two NEU1-specific siRNAs. Expression of NEU1, FN, α5, β1, Akt, and phosphorylated Akt was analyzed by Western blotting. Tubulin: loading control. Experiments were performed in triplicate, and representative blots are shown (**a**, **g**, **h**, **i**, **j**)

Sialidase activity and sialic acid expression were downregulated in YTS-1/NEU1, as expected (Fig. S4A, S4B). Lectin blotting assay showed clearly reduced binding capacity of YTS-1/NEU1 to lectin SNA and MAL-II (Fig. 4a). YTS-1/NEU1 showed a lower proportion of spindle cells (Fig. 4b), reduced adhesion to FN, laminin, and collagen V, and increased adhesion to Matrigel (Fig. S5). These findings indicate that cell adhesion properties were affected by alteration of cell surface sialylation.

Cell growth of YTS-1/NEU1 and T24/NEU1 was significantly slower than that of YTS-1/Ctrl and T24/Ctrl (Fig. 4c). Cell cycle assay revealed that NEU1 overexpression resulted in a significant arrest in G0/G1 phase (Fig. 4d, e). Flow cytometric analysis using the apoptosis markers annexin V and PI showed that NEU1-overexpressing cells were also likely to undergo apoptosis. Percentages of total apoptotic cells were 43.3% for T24/NEU1 vs. 19.85% for T24/Ctrl, and 20.52% for YTS-1/NEU1 vs. 3.94% for YTS-1/Ctrl (Fig. 4f). In addition, we found that cell cycle checkpoint regulators CDK2 and cyclin D1, which have been indicated to play important roles during G0/G1 cycle transition, were down-regulated, as well as the apoptosis makers such as cleaved Caspase 9 was up-regulated and anti-apoptotic protein Bcl-2 was down-regulated in YTS-1/NEU1 (Fig. 4g). In view of the findings that NEU1 overexpression decreased cell growth and increased cell apoptosis, we analyzed EMT marker protein expression in YTS-1/NEU1. NEU1-overexpressing YTS-1 cells showed no clear change of N-cadherin or vimentin expression (Fig. S6). In striking contrast, FN expression was significantly reduced in YTS-1/NEU1 (Fig. 4h).

### Akt signaling pathway is downregulated by NEU1 overexpression

FN, a high-molecular-weight glycoprotein found in extracellular matrix (ECM), binds to the membrane-spanning integrin α5β1 and helps mediate cell spreading and migration. Activation of α5β1 protects cells against apoptosis and stimulates cell growth through a phosphatidylinositol-3-kinase (PI3K)/Akt-dependent pathway [38]. In view of the downregulation of FN in NEU1-overexpressing cells **(**Fig. 4h**)**, we assessed expression of the α5β1 and Akt pathways in YTS-1/NEU1 and T24/NEU1. Expression of FN, α5, and β1 was lower in YTS-1/NEU1 than in YTS-1/Ctrl. Akt protein expression was similar in YTS-1/NEU1 and YTS-1/Ctrl, but Akt phosphorylation was clearly reduced in YTS-1/NEU1 **(**Fig. 4h**)**. Similarly, reduced expression of FN, α5, β1, and phosphorylated Akt was observed in T24/NEU1 relative to T24/Ctrl (Fig. 4e). We next treated YTS-1 with sialidase NeuA3, cloned from the non-pathogenic *Streptomyces avermitilis* and expressed in *E. coli* as described previously [39]. Expression of FN, α5, β1, and phosphorylated Akt was clearly reduced in the NeuA3-treated cells (Fig. 4i). Oppositely, knockdown of NEU1 expression in noncancerous HCV29 cells resulted in enhanced expression of FN, α5, and β1, and activation of Akt pathway (Fig. 4j). Taken together, these findings indicate that NEU1 overexpression reduces cell proliferation and enhances cell apoptosis through by downregulation of FN-integrin β1-mediated Akt signaling pathway.

### Alteration of FN-integrin α5β1 distribution and expression by NEU1 overexpression

**Fig. 5:**
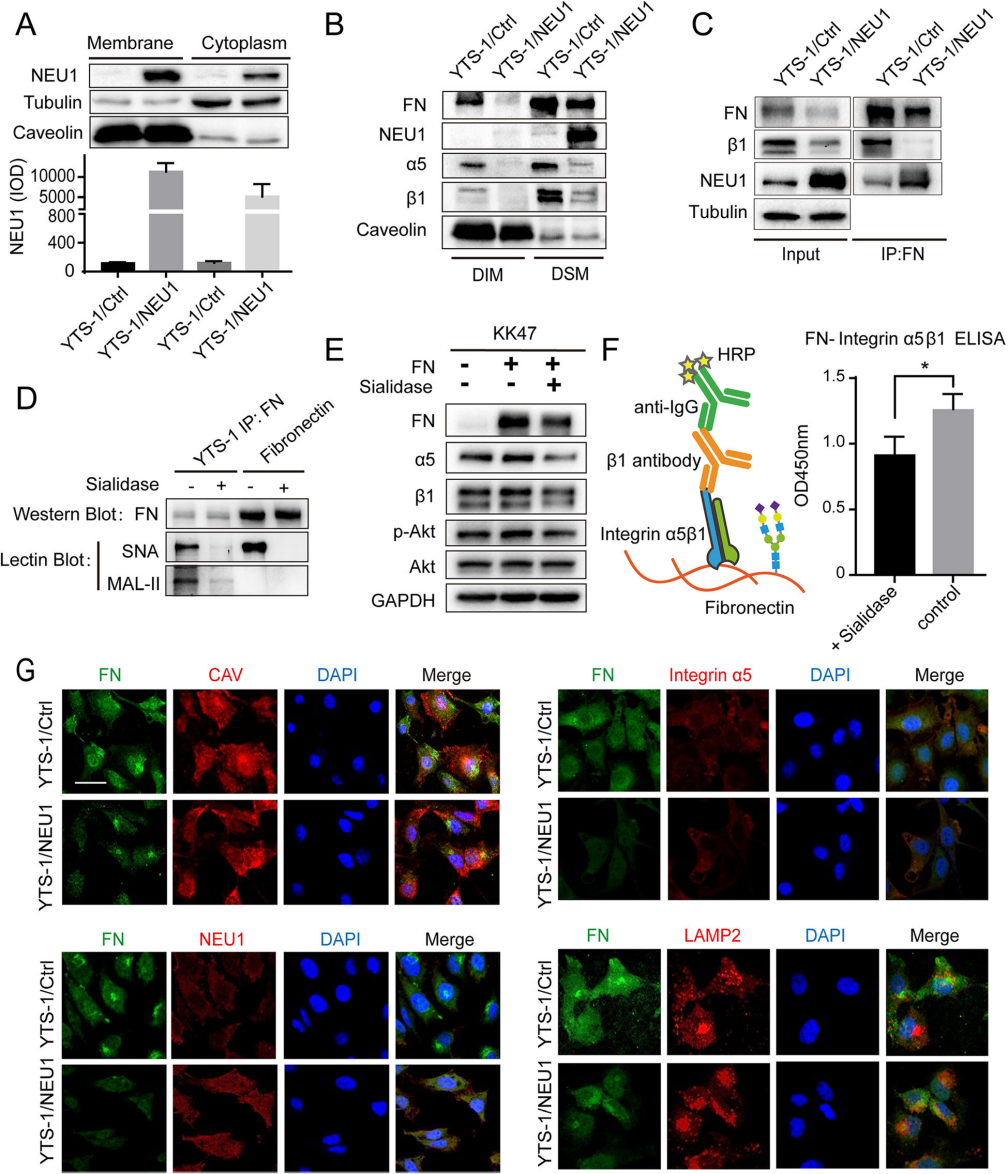
NEU1 alters α5β1 and FN distribution and expression on cell membrane. **a** Membrane and cytoplasmic proteins of YTS-1/Ctrl and YTS-1/NEU1 cells were extracted with a kit. NEU1 expression was analyzed by Western blotting and presented as IOD as in Fig. 2c. Tubulin was the cytoplasmic protein control and caveolin was the membrane control. **b** DIM and DSM proteins were extracted as described in Supplementary information/Methods, and expression of FN, NEU1, α5, and β1 was analyzed by Western blotting. Caveolin (caveolae marker of DIM) was used as reference. Representative results from two independent experiments are shown. **c** Co-IP assay: cells were lysed by modified RIPA buffer, incubated with anti-FN antibody (which binds to protein A/G Plus agarose) overnight at 4 °C with gentle rotation. FN, β1, and NEU1 in precipitate were assayed by Western blotting. Tubulin was used as loading control. **d** FN immunoprecipitated from YTS-1 cell lysate or purified from cells were analyzed by Western blotting and Lectin blotting. **e** FN (50 μg/mL) was coated on 12-well plates and treated with 1 U/mL sialidase for 1 h at 37 °C. KK47 cells (2 × 10^5^) were cultured on plates coated with normal FN and desialylated FN for 18 h, lysed with RIPA buffer, and subjected to SDS-PAGE and Western blotting. **f** Binding affinity between FN and integrin α5β1 was detected by modified ELISA method (see Methods). FN were fixed on to ELISA plates and treated with sialidase. Then integrin α5β1 were coated onto plates to interreact with FN. HRP conjugated anti-integrin β1 antibody and TMB-ELISA Substrate Solution were used to detect binding ratio. **g** YTS-1/Ctrl and YTS-1/NEU1 cells were seeded onto glass cover slips (diameter 12 mm) in 24-well tissue culture plates, fixed with 2% fresh paraformaldehyde/ PBS, blocked with 1% BSA/ PBS, and stained with anti-FN, anti-caveolin, anti-α5, anti-NEU1, or anti-LAMP2, as described in Supplementary information /Methods. For each antibody staining, clear DAPI staining of nuclei and merged photos are shown

NEU1 is usually localized in lysosomes, but can also be transported in extracellular vesicle or on the cell surface [17]. Assessment of NEU1 distribution showed that it was minimally expressed in YTS-1/Ctrl, but highly expressed in YTS-1/NEU1. In YTS-1/NEU1, a greater proportion of NEU1 was localized on cell membrane than in cytoplasm (Fig. 5a). Functions and downstream signaling pathways of integrin have been shown to be affected by compartmentalized expression on the cell surface. We therefore isolated proteins from plasma membrane detergent-soluble microdomain (DSM) and detergent-insoluble microdomain (DIM) fractions of YTS-1/Ctrl and YTS-1/NEU1, and examined the differential expression of NEU1, FN, α5, and β1 in the two fractions. In YTS-1/Ctrl, α5 and β1 were expressed primarily in DSM, and to a lesser degree in DIM. In YTS-1/NEU1, α5 and β1 expression was greatly reduced overall and almost nonexistent in DIM (Fig. 5b), while overexpressed NEU1 was localized mainly in DSM. FN was detected in both DIM and DSM in YTS-1/Ctrl, but exclusively in DSM in YTS-1/NEU1 **(**Fig. 5b**)**.

### NEU1 disrupts FN/ α5β1 interaction

To examine the effect of NEU1 on FN-integrin α5β1 interaction, we performed co-immunoprecipitation (co-IP) assay. β1 was clearly precipitated by FN in YTS-1/Ctrl, whereas no binding of β1 to FN was observed in YTS-1/NEU1 (Fig. 5c). NEU1 protein was detected in FN immunoprecipitation **(**Fig. 5c**)**, indicating that NEU1 is able to interact with FN. Sialylation of cell surface glycoproteins of cancer cells has been clearly shown to play an important role in malignant properties. FN has ~ 10 N-linked glycosylation and O-linked glycosylation sites [40], and sialic acid is always the terminal group in elongation of N-glycans and O-glycans. Sialylated and desialylated FN may therefore affect integrin binding and related downstream signaling. FN in YTS-1 cells was highly sialylated and the added sialidase could efficiently remove their sialic acids, the same as purified FN (Fig. 5d). FN expression is much lower in benign non-muscle-invasive bladder cancer KK47 than in YTS-1. We cultured KK47 cells on control plates (no FN), or on plates coated with FN or with sialidase-treated (desialylated) FN. Expression of α5, β1, and phosphorylated Akt was higher for cells on FN-coated plates than on control plates. In contrast, expression of these three components on desialylated-FN-coated plates was similar to that on control plates (Fig. 5e). Further, to investigate whether sialic acids on FN affected its affinity to integrin α5β1, an ELISA experiment was designed (Fig. 5f). Purified integrin α5β1 dimers showed lower binding affinity to desialylated FN, indicating that the sialic acid may change the binding ability of FN towards integrins in order to affect signal transduction.

The reduced expression of FN and α5 in YTS-1/NEU1 was confirmed by fluorescence microscopy (Fig. 5g). FN was clearly present in DSM of YTS-1/Ctrl, where it was co-localized with caveolin, but absent in DSM of YTS-1/NEU1. FN - NEU1 interaction was also observed in YTS-1/NEU1. FN showed an enhanced confocal signal with lysosome-associated membrane protein 2 (LAMP2), suggesting that loss of FN in YTS-1/NEU1 may result from degradation in lysosomes (Fig. 5g).

### Downregulation of NEU1 expression in bladder cancer as revealed by tissue microarray (TMA) analysis

**Fig. 6:**
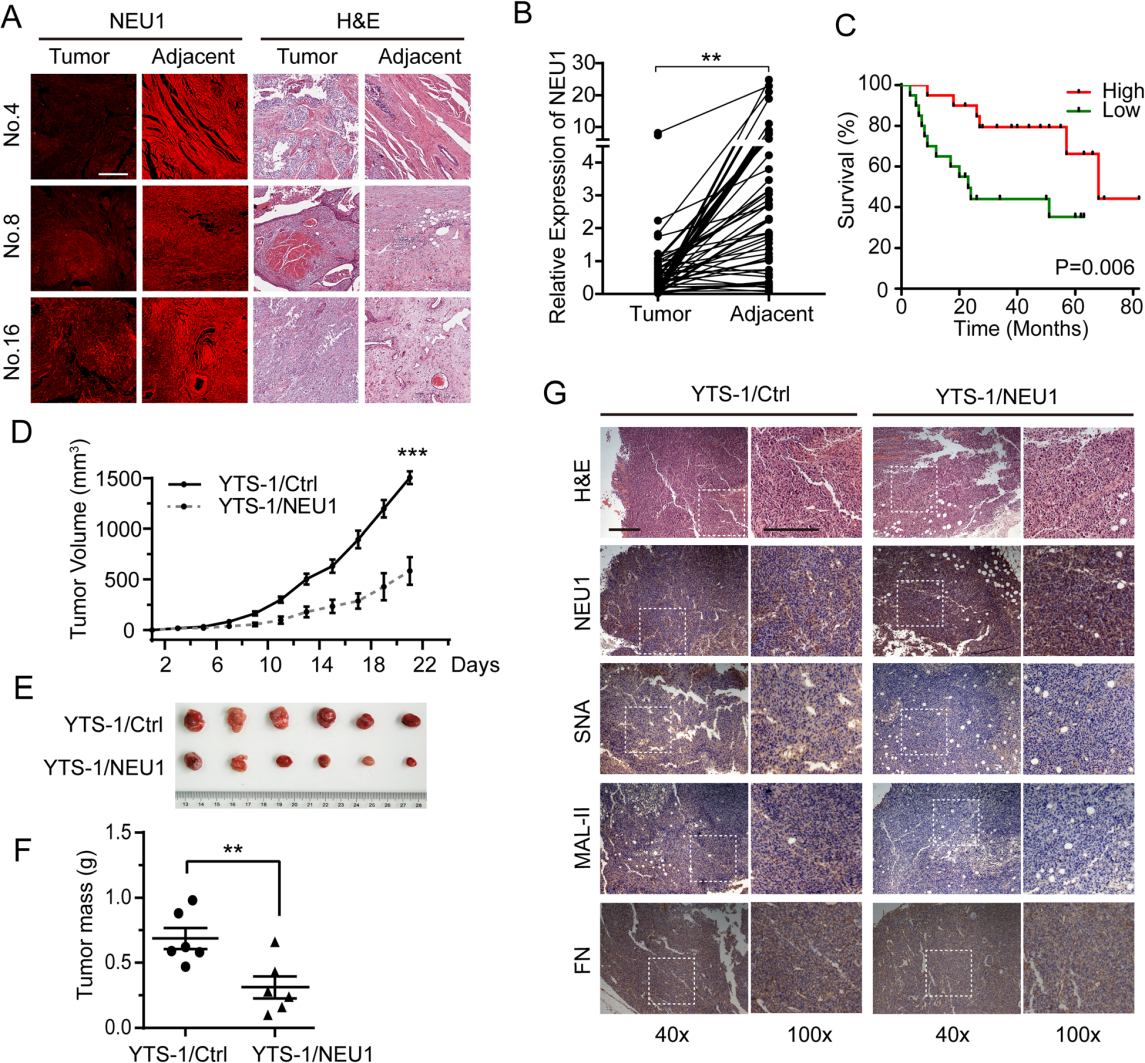
NEU1 overexpression suppresses tumor growth in vivo. NEU1 in bladder cancer clinical tissues was evaluated by immunostaining on tissue microarrays (TMAs). Each of the 44 TMA pairs consisted of two dots (one carcinoma tissue, two adjacent noncancerous tissues) from the same patient. Each pair was analyzed by anti-NEU1 antibody staining. **a** Representative NEU1 expression in carcinoma nest and adjacent noncancerous cells and hematoxylin and eosin (H&E) staining of samples 4, 8, and 16. Scale bar: 50 μm. **b** TMA scores from carcinoma tissue and matched noncancerous tissue for each pair were analyzed using the Image Pro Plus software program. **c** Kaplan-Meier overall survival curves for bladder cancer patients classified according to relative NEU1 expression level. TMAs from 55 patients were stained with anti-NEU1 antibody and scanned by GenePix microarray scanner. Patients were classified into “relatively high” (*n* = 20), “relatively low” (n = 20), and “non-significant” (*n* = 15) groups based on median NEU1 expression. **d** Tumor growth curve from BALB/c-nu mice injected with YTS-1/Ctrl or YTS-1/NEU1 cells. **e** Dissected tumors in nude mice (*n* = 6) injected with YTS-1/Ctrl or YTS-1/NEU1 cells, after 3 weeks. **f** Masses of dissected tumors. **g** Immunohistochemistry of tumor tissue from BALB/c-nu mice. Scale bar: 50 μm. Mean values were compared by paired Student’s t-test. *, *P* = 0.01–0.05. **, *P* = 0.001–0.005. ***, *P* < 0.001

NEU1 expression was evaluated using TMAs, including a total of 44 pairs of primary human bladder cancer tissue samples with matched adjacent noncancerous tissues (Table S3). NEU1 level was clearly higher in noncancerous bladder cells than in bladder cancer cells (Fig. 6a). In a summary of TMA analysis results, NEU1 expression was significantly lower in cancer tissue than in matched normal tissue for 40 of the 44 pairs (Fig. 6b). Survival analysis showed a significantly worse prognosis for patients with low (vs. high) NEU1 expression (Fig. 6c, Table S4). However, GEO data from 3 paired bladder cancer and adjacent normal tissues showed no significant difference of NEU1 expression at mRNA level (Fig. S7), indicating that the post-translational modification or degradation mechanism may involve in regulating NEU1 expression at protein level. The dysregulated expression of NEU1 in these clinical samples provides further evidence for a link between NEU1 expression and bladder tumor progression.

### NEU1 overexpression suppresses bladder tumor growth in vivo

To assess the importance of NEU1 in tumor formation in vivo, we injected YTS-1/Ctrl or YTS-1/NEU1 cells into BALB/c-nu mice and assayed tumor growth. The YTS-1/NEU1-injected mice showed significantly slower tumor growth (Fig. 6d), and a much smaller total tumor mass (Fig. 6e, f), in comparison to YTS-1/Ctrl-injected mice. In addition, immunohistochemistry of tumor tissue from BALB/c-nu mice injected with YTS-1/NEU1 cells showed significant higher NEU1 expression, lower sialic acid modification (stained by lectin SNA and MAL-II) and lower FN-integrin α5β1 expression, compared to the tumor from the mice injected with YTS-1/Ctrl cells (Fig. 6g). Moreover, in tumor tissues with overexpressed NEU1, less proliferation and more apoptosis were observed (Fig. S8). These findings are consistent with those from in vitro cell proliferation assay, and indicate that NEU1 overexpression suppresses bladder tumor growth in vivo*.*

## Discussion

During glycan synthesis, addition of sialic acid to terminal Gal or GalNAc acts as a termination signal and prevents further chain elongation. The sialylation state of glycoproteins and glycolipids is a critical factor modulating molecular recognition between adjacent cells, between cells and ECM, and between host cells and pathogens. Importantly, increased sialylation of cell surface molecules has been correlated with tumor aggressiveness [41]. Sialic acid receptor Siglec-15, which is broadly upregulated on human cancer cells, has been proved as a critical immune suppressor and as a potential target for normalization cancer immunotherapy [42]. Precise glycocalyx editing strategy which conjugated a sialidase to therapeutic monoclonal antibody trastuzumab cleaved the sialic acids on the cell surface and greatly increased tumor cell killing [43]. Sialylation state can be modified synergistically by sialyltransferases and sialidases, and there is considerable evidence for a role of aberrant sialyltransferase expression in cancer progression. However, far fewer studies have addressed the role of the four known sialidases in cancer progression.

As is known that NEU1 is a major endogenous sialidase that forms a high-molecular-weight multienzyme complex with protective protein/cathepsin A (PPCA) and the hydrolase β-galactosidase to protect them against rapid proteolytic degradation and facilitate their correct folding in lysosomes [44]. Interestingly, NEU1 was recently shown to be transported in vesicles and on the cell surface, and to perform on-site hydrolysis [45].

Published data on expression and function of NEU1 in various types of cancer appear somewhat contradictory. In human colon cancer, NEU1 expression was negatively correlated with metastasis [27]. In pancreatic cancer, enhanced NEU1 expression was involved in MMP9-EGFR signaling, and promoted cancer progression and metastasis [46]. In ovarian cancer, NEU1 siRNA inhibited cell proliferation, apoptosis, and invasion [47]. In the present study, downregulation of NEU1 was observed in bladder cancer cells and clinical samples, and NEU1 overexpression resulted in inhibition of proliferation, promotion of apoptosis, and inactivation of the Akt signaling pathway.

It is noteworthy here, that FN-integrin mediated signaling pathways, particularly the Akt pathway, have been well studied in numerous cell models [48, 49]. NEU1 has been shown to be associated with multiple receptor signaling complexes, including TOLL-like receptor [50] and growth factors PDGF-BB and IGF-2 [51]. In human airway epithelial cells, NEU1 was associated with substrates EGFR and MUC1, and regulated ligand-dependent EGFR autophosphorylation [52, 53]. Interestingly, we observed reduced levels of FN, α5, and β1 in both T24/NEU1 and YTS-1/NEU1. Present and previous observations, taken together, strongly indicate that overexpressed NEU1 inhibits the Akt pathway by disrupting FN-integrin α5β1 interaction.

**Fig. 7:**
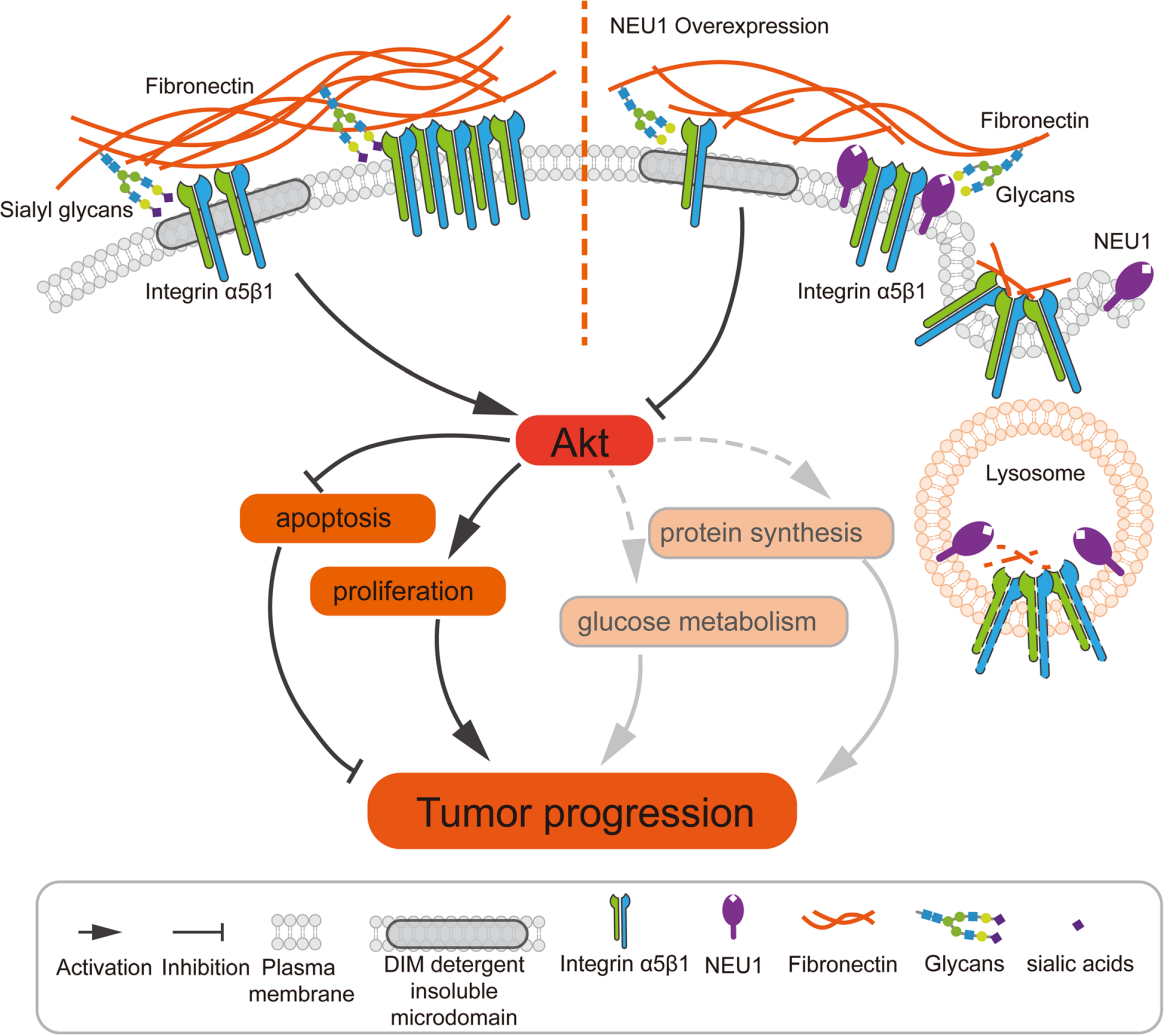
Model for NEU1 disrupting fibronectin-α5β1 interaction and inhibiting the Akt signaling pathway

Herein, we proposed that sialylation promotes the tight interaction FN-integrin α5β1, while de-sialylation reduces their binding capacity, and sialylation may further affect endocytosis/ recycling of FN and integrin (Fig. 7). Effects of altered sialylation of cell surface glycoproteins on malignant properties have been reported by many groups. Hypersialylation of β1 was shown to promote tumor progression by increasing adhesion to FN, collagen I, and certain ECM components in colonic adenocarcinoma [54] and human astrocytoma cells [55]. In contrast, removal of sialic acid from integrin O-glycans resulted in decreased integrin phosphorylation and inhibition of focal adhesion kinase (FAK) and ERK1/2 pathways [27, 56]. Sialylation of vitronectin, another ECM molecule, played a crucial role in activation of hepatic stellate cells during the liver regeneration process [57]. Although ~ 10 N-linked and O-linked glycosylation sites on FN have been identified, little is known regarding the effects of altered glycosylation on FN [40, 58]. It is important to elucidate the expression and function of sialylation on FN. We observed that NEU1 was distributed predominantly in the DSM area of the cell surface, and attracted FN to this area. NEU1 overexpression altered compartmental distribution of FN and integrin, and may disrupt their interaction. On the other hand, it is noted that glycosylation could affect endocytosis of membrane glycoproteins. Endocytosis of β1 plays an important role in matrix FN turnover, and β1 and FN appear to be trafficking together during endocytosis [59]. Following endocytosis, FN is trafficked and degraded by lysosomes [60]. Endocytosis of bisecting GlcNAc-modified EGFR and downstream ERK phosphorylation were both enhanced in N-acetylglucosaminyltransferase III (GnT-III)-overexpressing cells [61]. Sialylation of membrane glycoproteins is responsible for their intracellular accumulation and trafficking in some cases [62]. Lifespan and abundance of secreted proteins (mostly glycoproteins) depend on differentially exposed N-glycan structures, for which intrinsic NEU1 and other glycosidases provide useful models [63]. Overall sialylation state on the surface of NEU1-overexpressing cells was reduced in the present study. NEU1 overexpression also decreased FN and integrin β1 level of the plasma membrane, increased FN degradation by lysosomes, and consequently inhibited the downstream AKT pathway. This model is consistent with the observed effect of sialylation on endocytosis/ recycling of FN and integrin in bladder cancer cells. In accordance with our data, the decreased sialylation status may loose the interaction of FN and integrin α5β1 and further result in their endocytosis/ recycling. Therefore, a combined strategy involving experimental, computational, and structural biological approaches will help us evaluate the models described above.

## Conclusions

In conclusion, our observations indicate that NEU1 is an important modulator of the malignant properties of bladder cancer cells; i.e., NEU1 inhibited cancer cell proliferation, induced apoptosis, and suppressed tumor formation both in vitro and in vivo. NEU1 appeared to deactivate the Akt signaling pathway by disrupting FN-integrin α5β1 interaction. NEU1 is a potential therapeutic target for prognosis and treatment of bladder cancer. The detailed molecular mechanisms underlying the effects of NEU1 will be addressed in our subsequent study.

## Supplementary information

**Additional file 1. ** Supplementary information/Methods. (Cell lines and cell culture; Antibodies and reagents; Derivatization and separation of N-glycans; Derivatization and separation of N-glycans; Stable isotope labeling by amino acids in cell culture (SILAC); Semi-quantitative and quantitative real-time RT-PCR analysis; Cell transfection; NEU1 gene silencing (siRNA); Proliferation (MTT) assay; Western blotting; Lectin blotting; Immunofluorescence staining; Immunohistochemistry; Wound assay for motility; Cell adhesion assay; Determination of apoptosis by flow cytometry; Tissue microarray (TMA) analysis; Co-immunoprecipitation (co-IP); Extraction of detergent-soluble microdomain (DSM) and detergent-insoluble microdomain (DIM) fractions; TUNEL assay; Cell cycle assay). **Table S1.** Annotation and quantitative analysis by MALDI-TOF/TOF-MS of N-linked glycan. **Table S2.** Patient information for sialic acid lectin blot. **Table S3.** Patient information for analysis of NEU1 expression in tumor and adjacent tissues. **Table S4.** Patient information for survival analysis. **Fig. S1.** The intensity of NEU1 protein in LC-MS/MS analysis. **Fig. S2.***NEU1* mRNA expression in five bladder cancer or epithelial cell lines. **Fig. S3.** Cell motility during EMT. **Fig. S4.** Sialidase activity and sialic acid expression in NEU1-overexpressing cells. **Fig. S5.** Adhesion capacity of YTS-1/Ctrl and YTS-1/NEU1 cells. **Fig. S6.** EMT marker proteins in YTS-1/Ctrl and YTS-1/NEU1 cells. **Fig. S7.** NEU1 mRNA level in bladder cancer tissue. **Fig. S8**. Ki67 and TUNEL staining of mice tumor tissue.

## Data Availability

The materials and datasets used during the study are available from the corresponding author on reasonable request. This article contains Supplementary Information online.

## Abbreviations

4-MU-NANA: 2′-(4-methylumbelliferyl) -α-D-N-acetylneuraminic acid sodium
BSA: Bovine serum albumin
CDK2: Cyclin-dependent kinases 2
co-IP: co-immunoprecipitation
CV: Coefficient of variation
DIM: Detergent-insoluble microdomain
DMSO: Dimethyl sulphoxide.
DSM: Detergent-soluble microdomain
ECM: Extracellular matrix
EGFR: Epidermal growth factor receptor
ELISA: Enzyme-linked immunosorbent assay
EMT: Epithelial-mesenchymal transition
FBS: Fetal bovine serum
FN: Fibronectin
GnT-III: N-acetylglucosaminyltransferase III
HCV29: Normal bladder epithelia
HR LC-MS: High resolution liquid chromatography mass spectrometry
HRP: Horse radish peroxidase
J82: Transitional carcinoma urinary bladder
KK47: Benign non-muscle-invasive bladder cancer
LAMP2: Lysosome-associated membrane protein 2
MALDI-TOF: Matrix-Assisted Laser Desorption/ Ionization Time of Flight Mass Spectrometry
MAL-II: *Maackia amurensis* lectin, recognizing α2,3-linked sialic acids
MMP9: Matrix metalloprotein
MS: Mass spectrometry
NEU1: Sialidase-1 (neuraminidase 1)
Neu5Ac: N-acetylneuraminic acid
Neu5Gc: N-glycolylneuraminic acid
PBS: Phosphate buffered saline
PI3K: Phosphatidylinositol-3-kinase
PPCA: Protective protein/cathepsin A
RT-PCR: Quantitative real-time polymerase chain reaction
SILAC: Stable isotope labeling by amino acids in cell culture
siRNA: Small interfering RNA
SNA: *Sambucus nigra* lectin, recognizing α2,6-linked sialic acids
ST6GAL1: β-galactoside α-2,6-sialyltransferase 1
ST8SIA2: α-2,8-sialyltransferase 2
SV-HUC-1: Human uroepithelial cell line
T24: Transitional cancers of human urine bladder
TGFβ: Transforming growth factor β
TMA: Tissue microarray
YTS-1: Highly malignant invasive bladder cancer
