# Supplementary material for: Sialidase NEU1 suppresses progression of human bladder cancer cells by inhibiting fibronectin-integrin α5β1 interaction and Akt signaling pathway
Source: Cell Commun Signal. 2020 Mar 12;18:44. doi: 10.1186/s12964-019-0500-x (PMC7066847; doi:10.1186/s12964-019-0500-x)
Supplement: Supplementary file 2 — Additional file 1. Supplementary information/Methods. (Cell lines and cell culture; Antibodies and reagents; Derivatization and separation of N-glycans; Derivatization and separation of N-glycans; Stable isotope labeling by amino acids in cell culture (SILAC); Semi-quantitative and quantitative real-time RT-PCR analysis; Cell transfection; NEU1 gene silencing (siRNA); Proliferation (MTT) assay; Western blotting; Lectin blotting; Immunofluorescence staining; Immunohistochemistry; Wound assay for motility; Cell adhesion assay; Determination of apoptosis by flow cytometry; Tissue microarray (TMA) analysis; Co-immunoprecipitation (co-IP); Extraction of detergent-soluble microdomain (DSM) and detergent-insoluble microdomain (DIM) fractions; TUNEL assay; Cell cycle assay). Table S1. Annotation and quantitative analysis by MALDI-TOF/TOF-MS of N-linked glycan. Table S2. Patient information for sialic acid lectin blot. Table S3. Patient information for analysis of NEU1 expression in tumor and adjacent tissues. Table S4. Patient information for survival analysis. Fig. S1. The intensity of NEU1 protein in LC-MS/MS analysis. Fig. S2.NEU1 mRNA expression in five bladder cancer or epithelial cell lines. Fig. S3. Cell motility during EMT. Fig. S4. Sialidase activity and sialic acid expression in NEU1-overexpressing cells. Fig. S5. Adhesion capacity of YTS-1/Ctrl and YTS-1/NEU1 cells. Fig. S6. EMT marker proteins in YTS-1/Ctrl and YTS-1/NEU1 cells. Fig. S7. NEU1 mRNA level in bladder cancer tissue. Fig. S8. Ki67 and TUNEL staining of mice tumor tissue. [file 12964_2019_500_MOESM2_ESM.pdf]

Supplementary Information

Sialidase NEU1 suppresses progression of human bladder cancer cells by inhibiting fibronectin - integrin α5β1 interaction and the Akt signaling pathway

Xiaoman Zhou [1], Yanhong Zhai [1], Changmei Liu [1], Ganglong Yang [1], Jia Guo [1], Guang Li [2], Chengwen Sun [2], Xiaowei Qi [3], Xiang Li [4], Feng Guan [1, 4, *]

1. The Key Laboratory of Carbohydrate Chemistry and Biotechnology, Ministry of Education, School of Biotechnology, Jiangnan University, Wuxi, China;
2. Department of Urology, Affiliated Hospital of Jiangnan University, Wuxi, China;
3. Department of Pathology, Affiliated Hospital of Jiangnan University, Wuxi, China.
4. Provincial Key Laboratory of Biotechnology, Joint International Research Laboratory of Glycobiology and Medicinal Chemistry, College of Life Science, Northwest University, Xi'an, China;

[*] Corresponding author. E-mail: guanfeng@nwu.edu.cn

Keywords: sialic acids, sialidase, apoptosis, fibronectin, integrin

Methods

Cell lines and cell culture

HCV29 [1], KK47 [2], YTS-1 [3], and T24 cells were cultured in RPMI 1640 medium (HyClone; Logan, UT, USA) containing 10% fetal bovine serum (HyClone) and 1% penicillin/streptomycin (Gibco; Carlsbad, CA, USA). J82 cells were cultured in MEM medium (HyClone) containing 10% FBS and 1% penicillin/streptomycin. All cells were cultured at 37 °C in a 5% CO_2 atmosphere.

Antibodies and reagents

Antibodies used were: mouse anti-NEU1 IgG, mouse anti-N-cadherin IgG1, mouse anti-Cyclin D1 IgG (Santa Cruz Biotechnology; Santa Cruz, CA, USA), rabbit anti-FN IgG, mouse anti-vimentin IgG1, anti-β-tubulin (Sigma-Aldrich; St. Louis, MO, USA), mouse anti-integrin α5 IgG2a, rabbit anti-integrin β1 IgG, rabbit anti-Akt IgG, rabbit anti-phosphorylated-Akt IgG, rabbit anti-caveolin IgG, mouse anti-LAMP2 IgG, rabbit anti-Ki67 IgG, rabbit anti-Bcl-2 IgG, rabbit anti-caspase-9 IgG, rabbit anti-CDK2 IgG (Cell Signaling Technology; Boston, MA, USA), horseradish peroxidase (HRP)-conjugated goat anti-mouse IgG, and HRP-conjugated goat anti-rabbit IgG (Beyotime; Haimen, China).

TGFβ was from Sino Biological Inc (Beijing, China). Fibronectin was from Yeasen (Shanghai, China). Human integrin α5β1 protein were from Merck (Darmstadt, Germany). Lectins were from Vector Laboratories (Burlingame, CA, USA). FN, laminin, and collagen IV were from Sigma-Aldrich. Matrigel was from Corning Life Sciences (Tewksbury, MA, USA). Other reagents were from Sigma-Aldrich unless described otherwise.

Derivatization and separation of N-glycans

Sialylated N-linked glycans of glycoproteins were amidated by acetohydrazide and released in a size-exclusion spin ultrafiltration unit (Amicon Ultra-0.5 10 KD, Millipore; Bedford, MA, USA) as described previously [4]. In brief, 1 mg protein sample was denatured with 8 M urea, reduced, and alkylated by addition of 10 mM dithiothreitol (DTT) and 10 mM iodoacetamide (IAM). Samples were washed with 40 mM NH_4HCO_3 and desalted by deionized water. Desalted proteins were redissolved with 100 μL of 1 M acetohydrazide, 20 μL of 1 N HCl, and 20 μL of 2 M EDC, and the mixture was incubated at room temperature (RT) for 4 h. Amidated glycoproteins were washed with 40 mM NH_4HCO_3 and treated with PNGase F in 40 mM NH_4HCO_3 overnight at 37 °C. Glycans were centrifuged, lyophilized, and desalted with Sepharose 4B as described previously [5].

Stable isotope labeling by amino acids in cell culture (SILAC)

HCV29, KK47, and YTS1 cells were cultured in light, medium, or heavy isotope-labeled RPMI 1640 medium as described previously [6]. Total proteins from each cell line were extracted using T-PER Reagent (Thermo Scientific; San Jose, CA, USA) and mixed at 1:1:1. The mixture was digested in-solution as described previously [5]. In brief, proteins were reduced, alkylated by incubation with an equal amount of 10 mM DTT and 20 mM IAM, and digested by trypsin. LC-MS was performed using an LTQ Orbitrap mass spectrometer, and raw data were analyzed using the MaxQuant software program (v. 1.6.1.5, Max Planck Institute of Biochemistry, Germany) as described previously [7, 8].

Semi-quantitative and quantitative real-time RT-PCR analysis

RNAs were extracted using an RNApure Tissue Kit (CoWin Biotech; Beijing). RNA was reversed transcribed using ReverTra Ace-α (Toyobo; Shanghai, China). Primers for *NEU1* were

sense 5'-CATGATCATCGCTGAGGAGAT-3' and anti-sense 5'-TCTCCCTGGATATTGGCACT-3'. Primers for *β-actin* were sense *5*'-GGTCAAGCAGCATAATCCAAAG-3', anti-sense *5*'-CAAGGGCATAGCCTACCACAA-3'. Real-time RT-PCR was performed with thermocycle conditions 95 °C for 10 min, 40 cycles of 95 °C for 10 s and 60 °C for 1 min, in a 15-μL reaction system using UltraSYBR Mixture (CoWin Biotech). PCR products were analyzed using a CFX Manager (Bio-Rad; Hercules, CA, USA).

Cell transfection

Primers for *NEU1* were 5'-CCCAAGCTTATGACTGGGGAGCGACC-3' and 5'-GGAATTCCAGTGGCACAGCTCAGAGTG-3'. Following gel extraction, enzyme digestion, ligation, and transformation, *NEU1* gene was inserted into pcDNA3.1$^{(+)}$ plasmid (Thermo). YTS-1 cells transfected with NEU1 gene marked YTS-1/NEU1, the empty vector group as control marked YTS-1/Ctrl. Cells were cultured to 70-90% confluence in 12-well plates, and transfected with mixture of Lipofectamine 2000 Reagent (Thermo) and the constructed plasmid. Transfectants were isolated by growing in G418 (Thermo)-containing medium, and confirmed by Western blotting.

NEU1 gene silencing (siRNA)

Sequences of siRNA oligos for *NEU1*, synthesized by Invitrogen, were:

1#sense 5'-GCGAUGGAGCUUCAGCAAUTT-3',

1#anti-sense 5'-AUUGCUGAAGCUCCAUCGCTT-3';

2#sense 5'-GCUCCGGCAUUGUCUUCUUTT-3',

2#anti-sense 5'-AAGAAGACAAUGCCGGAGCTT-3'.

HCV29 cells were cultured to 90% confluence in 12-well plates in growth medium without

antibiotics, and transfected with siRNA oligos and Lipofectamine 2000. Transfection efficiency was determined by quantitative real-time RT-PCR and Western blotting.

Proliferation (MTT) assay

Cells were seeded in 96-well plates and incubated for 12, 24, 48, or 96 h. Each well was added with 4 μL MTT solution (Cers; Yantai, China) and incubated at 37 °C for 4 h. The reaction was terminated by addition of 100 μL DMSO, and absorbance at 595 nm was recorded immediately.

Western blotting

Cells were lysed with RIPA protein extraction reagent, centrifuged at 12,000 rpm for 15 min at 4 °C, and protein concentration was determined using a BCA kit (Beyotime). Proteins separated by SDS-PAGE were transferred onto PVDF membranes (Millipore). Membranes were blocked with 3% BSA in 0.1% Tween 20 TBS (10 mM Tris-HCl, 150 mM NaCl, pH 8.0), blotted with primary mAb, incubated with appropriate HRP-conjugated secondary antibody, and visualized using a Supersignal Chemiluminescence substrate kit (Thermo). Results were quantified using Gel-Pro Analyzer image analysis software (v4.0).

Lectin blotting

Cell or tissue samples were lysed, quantified, separated, and transferred to membranes as protocol above, blocked with 3% BSA in PBST (PBS with 0.05% v/v Tween), incubated with 1 μg/mL biotinylated lectins in 1% BSA with PBST at 4°C overnight. After washing three times with PBST, singles can be detected by ABC kit and viewed using chemiluminescence substrate kit as guidance.

Immunofluorescence staining

Cells were seeded onto sterilized 12 mm diameter glass coverslips in 24-well tissue culture plates, cultured to 70-90% confluence, washed with PBS, fixed with 2% fresh paraformaldehyde for 15 min at RT, permeabilized with 0.2% Triton X-100 in PBS for 10 min at RT, and blocked with 5% BSA in PBS overnight at 4 °C. For lectin staining, fixed cells were incubated with 20 μg/mL Cy3-labeled *Sambucus nigra* lectin (SNA) or *Maackia amurensis* lectin II (MAL-II) (Vector Laboratories; Burlingame, CA, USA) for 3 h at RT in the dark. For antibody staining, cells were incubated with primary antibody at 4 °C overnight, washed with PBS, incubated with FITC-labeled secondary antibody for 1 h at RT in the dark, further stained with DAPI for 10 min, and examined by fluorescence microscopy (model Eclipse E600; Nikon; Tokyo, Japan).

Immunohistochemistry

Tissues were dewaxed, and antigen retrieval was performed using citrate buffer (0.1 M citric acid and 0.1 M sodium citrate, pH 6.0). Slides were blocked [0.1% bovine serum albumin (BSA) and 0.5% Tween 20 with 10% normal goat serum] and incubated with the following primary antibodies overnight: anti-NEU1 (1:100), anti-FN (1:50), anti-integrin α5 (1:50), anti-integrin β1(1:100), anti-Ki67 (1:100), lectin SNA (1:100) and lectin MAL-II (1:100). Slides were incubated for 1 hour with HRP conjunct secondary antibodies or ABC Kit. Endogenous peroxidase was removed with 0.1% hydrogen peroxidase for 30 min. Antibody detection was performed using the DAB substrate. Slides were counterstained with H&E.

Wound assay for motility

Cells were cultured in 6-well plates with complete medium and grown to 100% confluence. Three separate wounds were scratched in each well using a 200 μL pipette tip. Wounds were

washed with PBS and added with serum-free culture medium to prevent cell proliferation. Wounds were photographed at 0 and 24 h.

Cell adhesion assay

96-well plates were coated with FN (1 μg/well), collagen IV (1 μg/well), matrigel (40 μg/well), or laminin (1 μg/well) at 37 °C for 2 h, rinsed and blocked with 1% BSA in HBSS at 37 °C for 1 h. Cells were cultured in serum-free medium 8 h, and seeded into 96-well plates (4×10^4 cells/ well). After 30 min incubation at 37 °C, nonadherent cells were gently removed with HBSS. Adherent cells were fixed with 2% fresh paraformaldehyde for 10 min and stained with 0.1% crystal violet in 20% methanol for 10 min. Excess dye was washed off with PBS. Crystal violet in the cells was dissolved in 100 μL of 10% acetic acid, and absorbance was measured at 595 nm.

Determination of apoptosis by flow cytometry

Apoptotic cells were quantified using an Annexin V-FITC detection kit (Beyotime) according to the manufacturer's protocol. In brief, cells were collected and washed in cold PBS, resuspended in 1× binding buffer in the presence of Annexin V-FITC and PI, incubated for 20 min at RT in the dark, and subjected to flow cytometry (BD Biosciences; Franklin Lakes, NJ, USA). Data were analyzed using the FlowJo software program (Tree Star; San Carlos, CA, USA).

Tissue microarray (TMA) analysis

Bladder cancer TMAs consisting of 44 cases of bladder carcinoma tissue with matched adjacent normal bladder tissue were from Shanghai Outdo Biotech Co. Nonspecific protein on slides was blocked by incubation with 5% BSA in PBS at 4 °C overnight. Specific antibodies were applied to slides and incubated for 3 h in the dark. Slides were scanned with a confocal scanner

and photographed under fluorescence microscopy as above. Staining intensity of TMAs was evaluated and scored by automated quantitative imaging using the Image Pro Plus software program (Media Cybernetics; Carlsbad, CA, USA).

Co-immunoprecipitation (co-IP)

Cells were washed three times with ice-cold PBS and added with ice-cold weak RIPA (Beyotime). Lysates were incubated on ice for 30 min, and centrifuged at 10,000 x *g* for 10 min at 4 °C. One mL supernatant was transferred to a microcentrifuge tube, added with primary antibody, and incubated 1 h at 4° C. The mixture was added with 20 μL resuspended Protein A/G PLUS-Agarose (Santa Cruz Biotechnology), rotated at 4 °C overnight, and centrifuged at 1000 × *g* for 5 min at 4 °C. Supernatant was aspirated and discarded. The pellet was washed with PBS and resuspended in 40 μL sample buffer. Proteins were released by boiling for 10 min and collected by centrifugation.

Extraction of detergent-soluble microdomain (DSM) and detergent-insoluble microdomain (DIM) fractions

DSM and DIM fractions were extracted as described previously [9]. In brief, cells were harvested by trypsinization, centrifuged, washed three times with ice-cold PBS, resuspended in buffer A (25 mM MES, 150 mM NaCl [pH 6.5]), and added with an equal volume of buffer B (25 mM MES, 150 mM NaCl [pH 6.5], 2% Triton X-100 (or NP40), 1% protease inhibitor cocktail). Lysates were incubated on ice for 30 min. Insoluble fractions were centrifuged at 13,000 × *g* for 30 min at 4 °C. Supernatant (DSM) was collected, and the insoluble pellet was resuspended in buffer C (1% Triton X- 100, 10 mM Tris-HCl [pH 7.6], 500 mM NaCl, 1% protease inhibitor cocktail, 60 mM octyl glucoside) for 30 min on ice. Debris was pelleted by centrifugation at 13,000

× *g* for 30 min at 4 °C, and supernatant (DIM) was collected.

TUNEL assay

TUNEL assay was performed using a Roche TUNEL Kit (Roche, Basel, Switzerland) following the manufacturer's instructions. Briefly, slides were rehydrated with graded ethanol, and incubated with proteinase K (20 μg/ml). Following digestion, the labeling reaction was ended by adding rTdT reaction mix for 1 h at 37°C in a humidified chamber. The rTdT enzyme reaction was terminated by stopping buffer. Slides were washed twice with PBS. Endogenous peroxidase was blocked with 0.3% hydrogen peroxide in PBS. After washing with PBS, streptavidin-HRP solution was added onto slides and incubated in a humidified chamber at room temperature. Excess unbound streptavidin-HRP was removed by PBS. Chromogenic substrate 3,3' -diaminobenzidine (DAB) solution was added. The reaction was stopped by rinsing the slides with deionized water. Finally, testicular nuclei were counterstained with hematoxylin.

Cell cycle assay

Cells were harvested and suspended in 0.1% BSA/PBS. Cells were fixed with 70% EtOH and washed with PBS. RNAs were digested using RNase and DNAs were stained with PI in the dark. Finally, cells were washed with PBS, resuspended, and subjected to FACS analysis. Data were analyzed using NovoExpress (ACEA, China) with Watson model.

References

1. Masters JRW, Hepburn PJ, Walker L, Highman WJ, Trejdosiewicz LK, Povey S, Parkar M, Hill BT, Riddle PR, Franks LM: Tissue Culture Model of Transitional Cell Carcinoma Characterization of Twenty-two Human Urothelial Cell Lines. Cancer Research 1986, 46:8.
2. H. H, M. K, K. N, T. K, K. T, K. N, K. K, F. M: Established cell line of urinary bladder carcinoma (KK-47): growth, heterotransplantation, microscopic structure and chromosome pattern. Jpn J Urol 1979, 70:10.
3. H. K, K. N, K. S: Establishment of a new cell line (YTS-1) derived from a human urinary bladder carcinoma and its characteristics. Jpn J Urol 1986:6.
4. Gang-Long Y, Tian-Ran M, Zheng L: Enrichment and Characterization of Total N-linked Glycans From Glycoproteins by Ultrafiltration Units and Mass Spectrometry. Progress in Biochemistry and Biophysics 2014, 41:6.
5. Yang G, Cui T, Wang Y, Sun S, Ma T, Wang T, Chen Q, Li Z: Selective isolation and analysis of glycoprotein fractions and their glycomes from hepatocellular carcinoma sera. Proteomics 2013, 13:1481-1498.
6. Bendall SC, Hughes C, Stewart MH, Doble B, Bhatia M, Lajoie GA: Prevention of amino acid conversion in SILAC experiments with embryonic stem cells. Mol Cell Proteomics 2008, 7:1587-1597.
7. Washburn MP, Wolters D, Yates JR, 3rd: Large-scale analysis of the yeast proteome by multidimensional protein identification technology. Nat Biotechnol 2001, 19:242-247.
8. Cox J, Mann M: MaxQuant enables high peptide identification rates, individualized p.p.b.-range mass accuracies and proteome-wide protein quantification. Nat Biotechnol 2008, 26:1367-1372.
9. Solomon KR, Mallory MA, Finberg RW: Determination of the non-ionic detergent insolubility and phosphoprotein associations of glycosylphosphatidylinositol-anchored proteins expressed on T cells. Biochem J 1998, 334 (Pt 2):325-333.

Table S1: Annotation and quantitative analysis by MALDI-TOF/TOF-MS of N-linked glycan structures in HCV29, SV-HUC-1, KK47, YTS-1, and J82 cells.

**Experimental Spectrum m/z**			**Glycan Structure**[a]	**Average Intensity Ratio**			
Spe 1	Spe 2	Distance		KK47/ SV-HUC-1	KK47/ HCV29	YTS1/ KK47	J82/ KK47
1496.352	1502.368	6.016	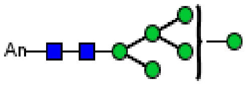	0.70527	2.70816	—[b]	0.84349
1515.797	1521.787	5.990	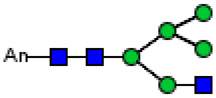	—	1.26696	—	—
1658.566	1664.575	6.009	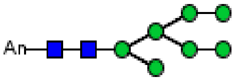	0.69004	1.45285	—	0.61537
1780.747	1786.753	6.007	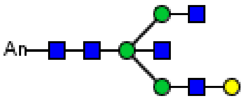	0.73180	1.69973	—	0.56751
1802.638	1808.647	6.009	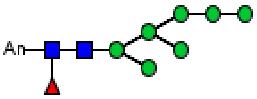	0.72973	1.72398	0.67283	0.57722
1820.597	1826.598	6.000	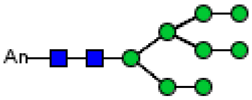	0.67991	1.64941	0.49048	0.52145
1833.495	1839.431	6.020	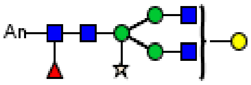	0.64159	—	1.34246	—
1839.431	1845.501	6.07	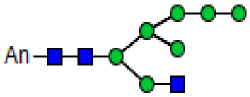	0.74416	—	—	—
1887.045	1893.108	6.063	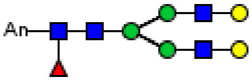	—	—	—	1.50053

Experimental Spectrum m/z			Glycan Structure[(a)]	Average Intensity Ratio			
Spe 1	Spe 2	Distance		KK47/ SV-HUC-1	KK47/ HCV29	YTS1/ KK47	J82/ KK47
1900.817	1906.842	6.025	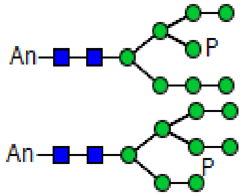	—	—	1.84865	—
1942.512	1948.515	6.003	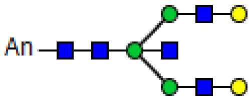	0.89985	—	1.29428	0.69404
1964.697	1970.700	6.003	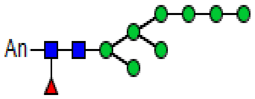	0.81122	—	1.10907	0.61209
1982.639	1988.642	6.003	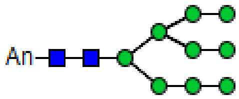	0.84666	2.13306	1.10635	0.70754
1996.795	2002.813	6.019	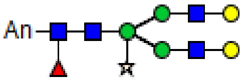	0.99966	—	1.17149	0.45996
2032.498	2038.546	6.048	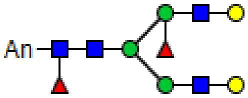	0.59239	—	—	0.35327
2062.878	2068.793	5.914	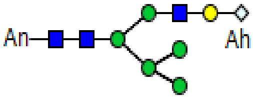	—	—	3.02792	—
2106.152	2112.140	5.988	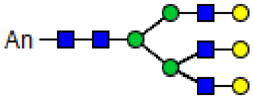	—	1.34259	—	0.75909
2125.474	2131.498	6.025	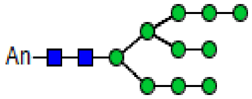	0.65879	1.05046	—	0.43283
2144.799	2150.800	6.001	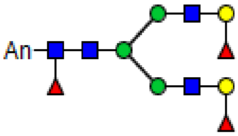	0.57053	2.38555	0.86671	0.63474

Experimental Spectrum m/z			Glycan Structure[a]	Average Intensity Ratio			
Spe 1	Spe 2	Distance		KK47/ SV-HUC-1	KK47/ HCV29	YTS1/ KK47	J82/ KK47
2178.777	2184.781	6.004	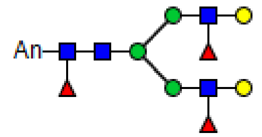	0.38622	—	—	—
2194.109	2200.107	5.998	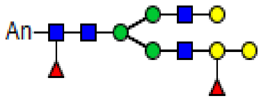	—	—	—	—
2224.939	2230.955	6.016	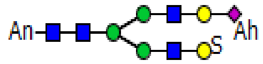	—	—	3.54425	—
2233.648	2239.658	6.010	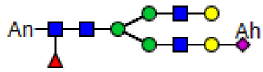	2.63599	—	—	1.06230
2251.914	2257.915	6.001	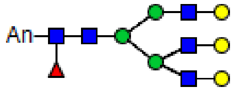	5.52974	0.82616	0.44699	1.08668
2266.433	2272.520	6.086	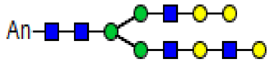	—	—	—	—
2293.431	2299.291	5.86	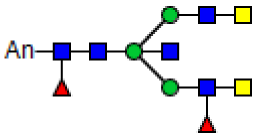	—	0.66924	—	—
2379.643	2385.648	6.005	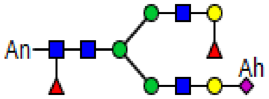	0.36052	0.97452	—	—
2386.979	2392.992	6.013	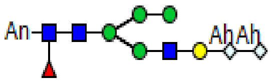	—	—	3.46592	—
2471.257	2477.259	6.002	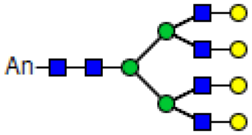	1.84471	1.17369	1.02459	1.04630

Experimental Spectrum m/z			Glycan Structure[(a)]	Average Intensity Ratio			
Spe 1	Spe 2	Distance		KK47/ SV-HUC-1	KK47/ HCV29	YTS1/ KK47	J82/ KK47
2519.706	2525.777	6.071	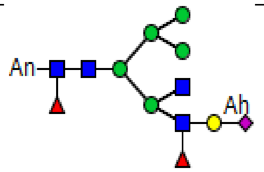	3.02640	—	—	—
2549.017	2555.095	6.078	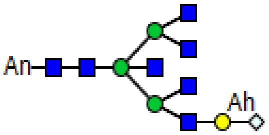	—	—	3.71565	—
2580.965	2586.975	6.010	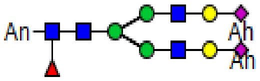	1.35267	—	—	1.03214
2599.028	2605.034	6.005	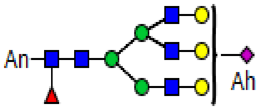	2.65444	0.94128	0.87760	0.78495
2616.905	2622.904	5.999	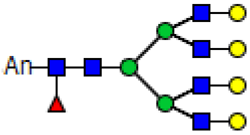	6.53986	1.15237	0.67631	0.73059
2632.539	2638.530	6.020	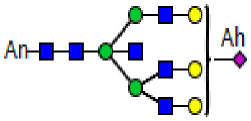	—	—	0.97911	—
2672.309	2678.249	5.940	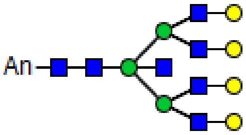	—	0.65989	—	—
2683.492	2689.535	6.043	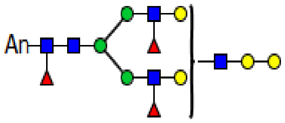	—	0.98409	—	0.91940
2711.129	2717.201	6.072	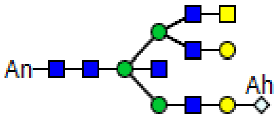	—	—	4.15462	—

Experimental Spectrum m/z			Glycan Structure[(a)]	Average Intensity Ratio			
Spe 1	Spe 2	Distance		KK47/ SV-HUC-1	KK47/ HCV29	YTS1/ KK47	J82/ KK47
2818.209	2824.245	6.037	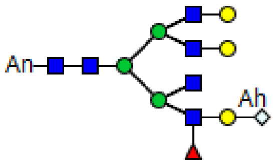	—	0.82616	1.59610	—
2873.001	2879.031	6.030	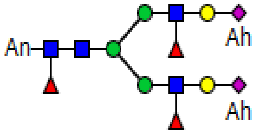	—	—	5.01091	—
2946.221	2952.235	6.015	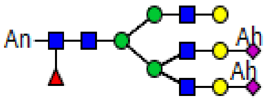	1.71386	—	1.07359	0.38150
2964.201	2970.214	6.014	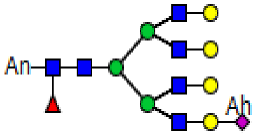	2.71043	—	1.16402	0.55699
2982.330	2988.302	5.971	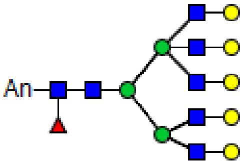	—	—	—	3.29718
3031.395	3037.418	6.023	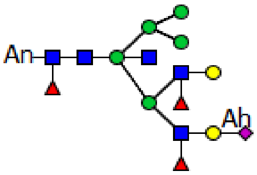	—	—	—	—
3035.134	3041.199	6.065	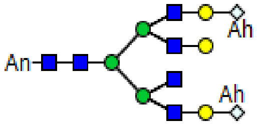	—	0.94128	0.16319	—
3146.946	3152.934	5.988	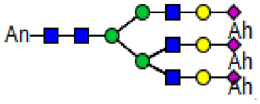	0.42273	—	—	—
3292.995	3298.986	5.991	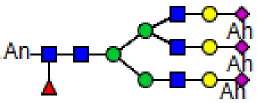	1.23957	—	—	0.99725

Experimental Spectrum m/z			Glycan Structure[(a)]	Average Intensity Ratio			
Spe 1	Spe 2	Distance		KK47/ SV-HUC-1	KK47/ HCV29	YTS1/ KK47	J82/ KK47
3310.948	3316.855	5.907	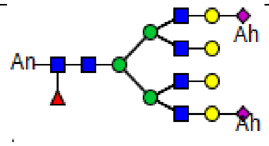	0.97370	1.95405	1.74017	0.87286
3329.376	3335.379	6.003	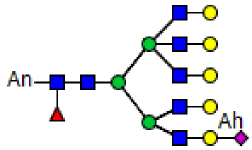	—	—	0.47023	0.97292
3347.222	3353.240	6.017	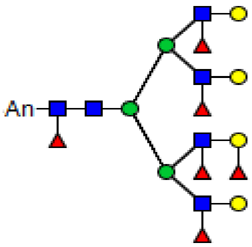	—	—	—	1.77904
3353.653	3359.823	6.170	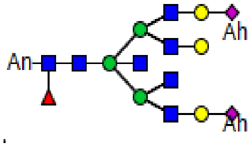	—	—	4.91082	—
3659.270	3665.113	5.843	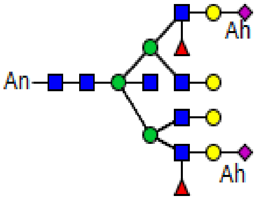	—	—	—	—

(a) Monosaccharides are represented according to MS-tools from EUROCarbDB (GlcNAc, ■; Man, ●; Gal, ○; Fuc, ▲; Neu5Ac, ◆; Neu5Gc, ◇; Xyl, ☆.) (b) not detected in cell pools.

Table S2. Patient information for sialic acid lectin blot

Patient ID	Age	Gender	T stage
142494	65	male	T3
180648	69	male	T3
163136	51	male	T2
159652	59	male	T3
161573	70	male	T3
161877	60	male	T3

Table S3. Patient information for analysis of NEU1 expression in tumor and adjacent tissues.

Characteristic	Bladder cancer (n=44)
Gender	
Male	29
Female	15
Age at diagnosis	
≥ 64	21
＜64	23
T stage	
T1-T2	18
T3-T4	26
TNM stage	
I	4
II	11
III	21
IV	8

Table S4. Patient information for survival analysis.

Characteristic	Bladder cancer (n=56)
Gender	
Male	47
Female	9
Age at diagnosis	
≥ 64	39
＜64	17
T stage*	
Tis	5
T1-T2	25
T3-T4	24
TNM stage*	
I	15
II	12
III	20
IV	7

* Information for two patients is missing.

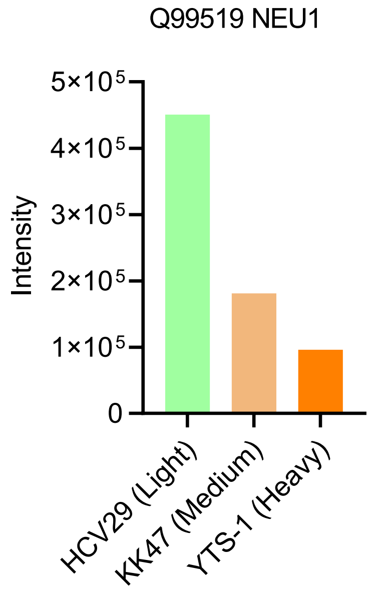

Figure S1. The intensity of NEU1 protein in LC-MS/MS analysis. The normalized intensity of NEU1 protein in HCV29 (Light label), KK47 (Medium label) and YTS-1 (Heavy label).

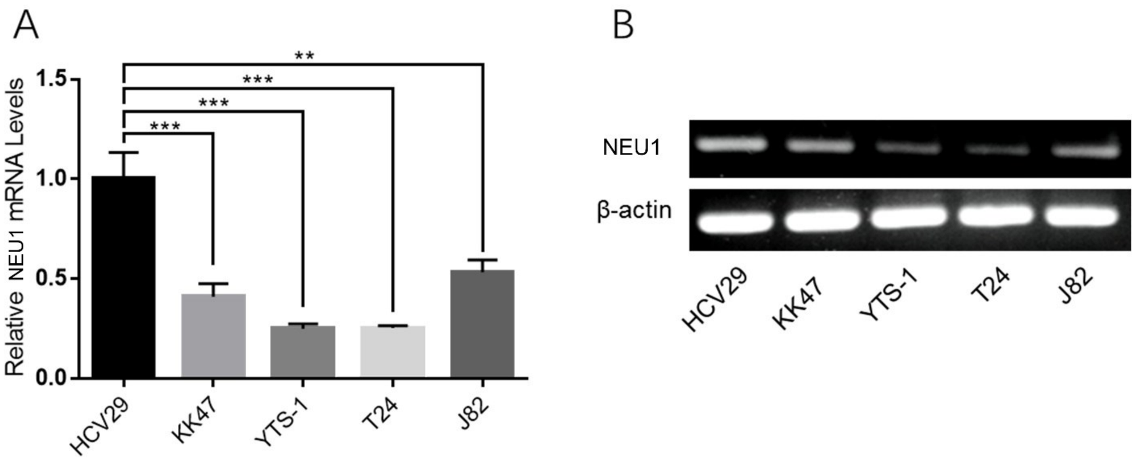

Figure S2. *NEU1* mRNA expression in five bladder cancer or epithelial cell lines. RNAs from HCV29, KK47, YTS-1, J82, and T24 cells were extracted and reversed transcribed. *NEU1* expression at the mRNA level was determined by real-time RT-PCR (**A**) and semi-quantitative PCR (**B**). **, P= 0.001- 0.005. ***, P< 0.001.

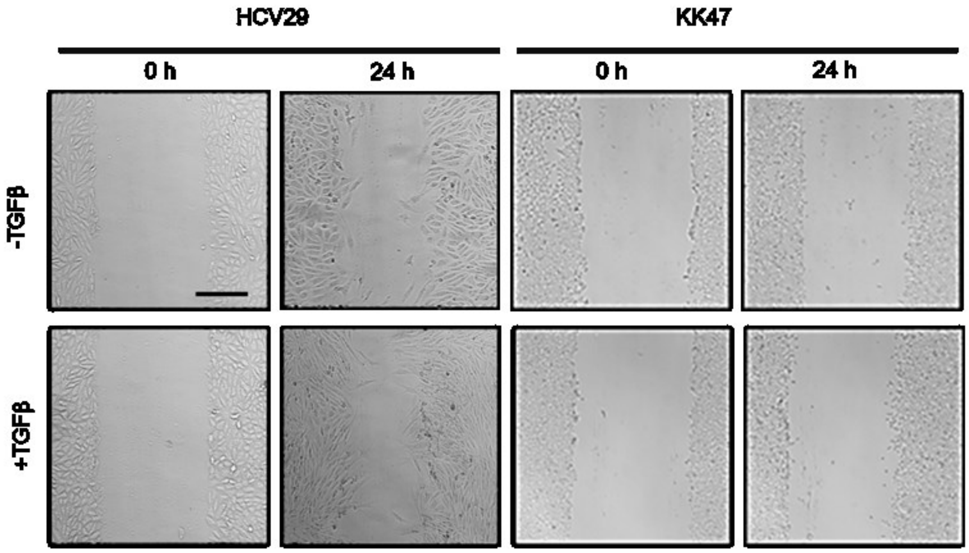

Figure S3. Cell motility during EMT. HCV29 and KK47 cells were cultured in 6-well plates with complete medium and treated with 5 ng/mL TGFβ for 24 h. Wounds were created and processed as described in Methods.

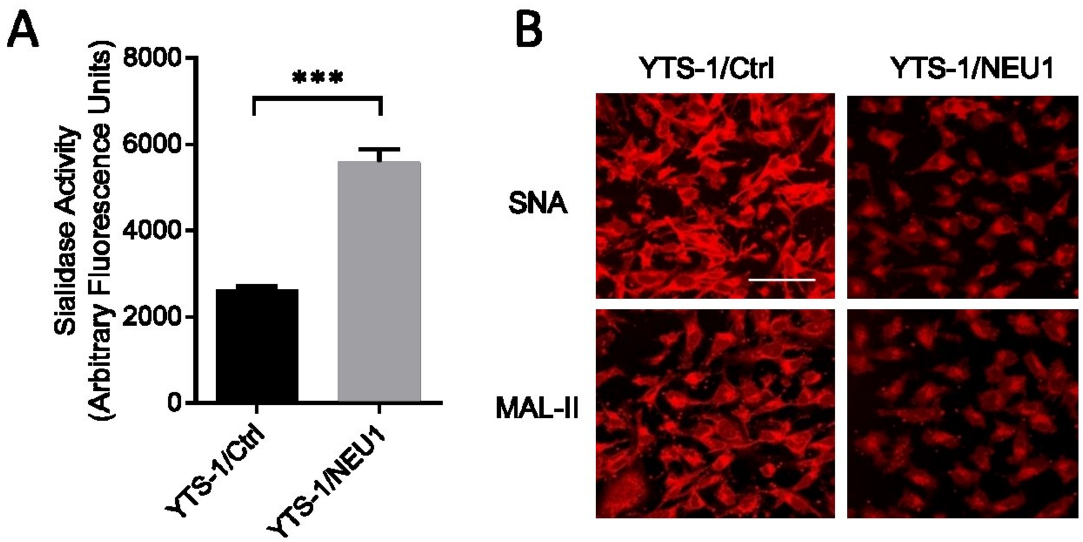

Figure S4. Sialidase activity and sialic acid expression in NEU1-overexpressing cells.

(A) Sialidase activity of YTS-1/Ctrl and YTS/NEU1 cells. 1×10^6 cells were suspended in 500 mM sodium acetate containing 0.1% Triton X-100 and protease inhibitor, and incubated for 1 h at 37 °C. The reaction was terminated by addition of an equal volume of glycine/ NaOH buffer. Fluorescence intensity was measured by spectrofluorometer with excitation wavelength 365 nm and emission wavelength 450 nm, and presented as mean ± SD from triplicate experiments. ***, $P< 0.001$. (**B**) YTS-1/Ctrl and YTS/NEU1 cells were incubated with Cy3-labeled SNA and MAL-II, and examined by fluorescence microscopy as described in Methods. Scale bar: 100 μm.

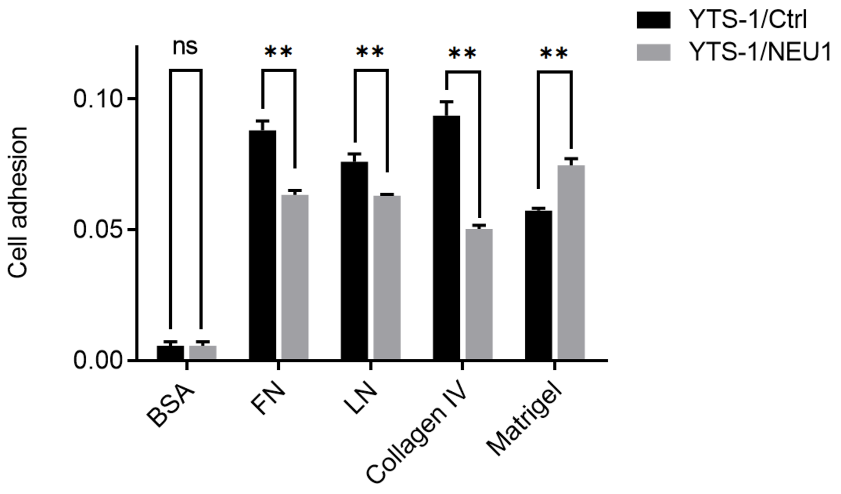

Figure S5. Adhesion capacity of YTS-1/Ctrl and YTS-1/NEU1 cells. Adhesion capacity to various ECM molecules (FN, laminin (LN), collagen IV, Matrigel) was determined by cell adhesion assay as described in Methods. Data were analyzed by the Prism software program, and presented as mean ± SD from triplicate experiments. **, P= 0.001-0.005. NS, not significant.

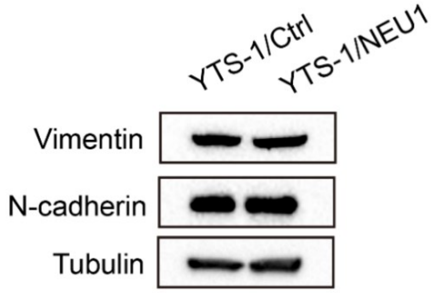

Figure S6. EMT marker proteins in YTS-1/Ctrl and YTS-1/NEU1 cells. Total proteins were extracted and subjected to SDS-PAGE and Western blotting. Signals for the EMT marker proteins vimentin and N-cadherin were detected using Supersignal Chemiluminescence substrate kit.

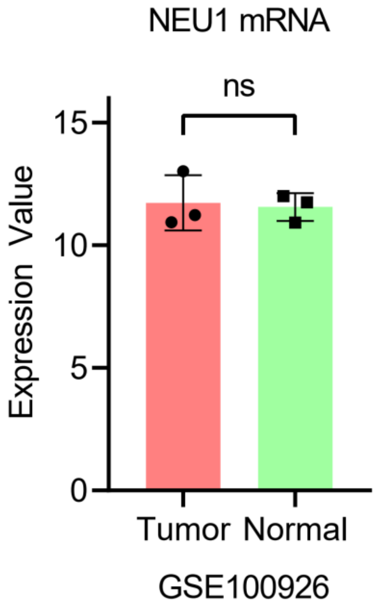

Figure S7. NEU1 mRNA level in bladder cancer tissue. NEU1 mRNA level was quantified in GEO dataset GSE100926.

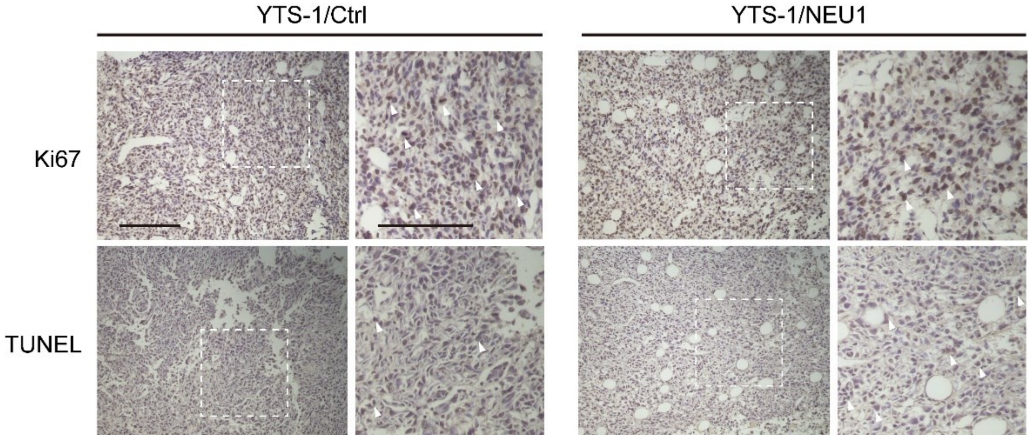

Figure S8. Ki67 and TUNEL staining of mice tumor tissue. Ki67 and TUNEL staining were performed as described in Supplementary Information / Methods. The positive staining was marked with white arrow in the enlarged pictures.
